# Genomic analysis of Asian honeybee populations in China reveals evolutionary relationships and adaptation to abiotic stress

**DOI:** 10.1002/ece3.6946

**Published:** 2020-11-02

**Authors:** Peng Shi, Jun Zhou, Huali Song, Yujuan Wu, Lan Lan, Xiangyou Tang, Zhengang Ma, Charles R. Vossbrinck, Bettina Vossbrinck, Zeyang Zhou, Jinshan Xu

**Affiliations:** ^1^ College of Life Sciences Chongqing Normal University Chongqing China; ^2^ Engineering Research Center of Biotechnology for Active Substances Ministry of Education Chongqing China; ^3^ State Key Laboratory of Silkworm Genome Biology Southwest University Chongqing China; ^4^ Department of Environmental Science Connecticut Agricultural Experiment Station New Haven CT USA; ^5^ Department of Math and Sciences Gateway Community College New Haven CT USA

**Keywords:** adaptation, *Apis cerana*, evolution, population, signal induction, stress

## Abstract

The geographic and biological diversity of China has resulted in the differential adaptation of the eastern honeybee, *Apis cerana*, to these varied habitats. *A. cerana* were collected from 14 locations in China. Their genomes were sequenced, and nucleotide polymorphisms were identified at more than 9 million sites. Both STRUCTURE and principal component analysis placed the bees into seven groups. Phylogenomic analysis groups the honeybees into many of the same clusters with high bootstrap values (91%–100%). Populations from Tibet and South Yunnan are sister taxa and together represent the earliest diverging lineage included in this study. We propose that the evolutionary origin of *A. cerana* in China was in the southern region of Yunnan Province and expanded from there into the southeastern regions and into the northeastern mountain regions. The Cold‐Temperate West Sichuan Plateau and Tropical Diannan populations were compared to identify genes under adaptive selection in these two habitats. Pathway enrichment analysis showing genes under selection, including the Hippo signaling pathway, GABAergic pathway, and trehalose‐phosphate synthase, indicates that most genes under selection pressure are involved in the process of signal transduction and energy metabolism. qRT‐PCR analysis reveals that one gene under selection, the *AcVIAAT* gene, involved in the GABAergic pathway, is responding to cold temperature stress. Through homologous recombination, we show that the *AcVIAAT* gene is able to replace the *CNAG_*01904 gene in the fungus *Cryptococcus neoformans* and that it makes the fungus less sensitive to conditions of oxidative stress and variations in temperature. Our results contribute to our understanding of the evolutionary origin of *A. cerana* in China and the molecular basis of environmental adaptation.

## Graphical abstract

We identified the Asian honeybee as seven genetic lineages and clarified their genetic relationship and then proposed that the origin of Chinese honeybee in the southern Asian geographic region including south Tibet, south Yunnan, and Himalaya–Hengduan Mountains. We found many genes in response to the abiotic stress were under selection for the genetic population in the regions of higher altitudes. Among these selective genes, based on homologous recombination in *C. neoformans*, we firstly clarified the gene involved in the pathway of transporting gamma‐aminobutyrate has contribution to tolerate the abiotic stress.

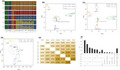

## 1 INTRODUCTION

Honeybees are economically important insects due to their role in honey production and agricultural pollination. The Asian honeybee *Apis cerana* (also referred to as the Eastern honeybee) is one of ten honeybee species comprising the genus *Apis* and has a wide geographic distribution in Asia (Arias & Sheppard, 2005; Engel & Schultz, 1997; Willis et al., 1992). Previous studies have focused on the morphological diversity of *A. cerana* based on morphometric clustering (Damus & Otis, 1997; Hepburn et al., 2001; Radloff et al., 2010; Radloff, Hepburn, Fuchs, 2005; Ruttner, 1988; Tan et al., 2008). In an early study, *A. cerana* was grouped into four main clusters. The Chinese honeybees together with native honeybees from Afghanistan, Pakistan, North India, and North Vietnam were placed in one group (Ruttner, 1988). Chinese honeybees have been classified into five clusters based on the morphometric data from samples across the country, including Hainan Island, East, Tibet, Diannan, and Aba groups (Yang, 2001). Another morphometric analysis of Asian *A. cera*na has shown six main morphoclusters and places the *A. cera*na in China in both morphoclusters I and II (Radloff, Hepburn, Fuchs, Otis, et al., 2005), which shows that *A. cera*na in China is composed of four subclusters: Aba, Tibet, central/eastern China, and southern China. A more recent study recognizes nine morphological and ecological types of Chinese honeybees (Aba, Diannan, Tibet, Yun‐Gui plateau, North China, Southern China, Changbaishan, Hainan, and Central China) based on morphological features and geographic location (Ge et al., 2011). These studies, showing the high degree of morphological differentiation among populations of Chinese honeybees, highlight the need for a larger data set available through comparative molecular analysis. Initial molecular studies comparing mtDNA or ssrDNA with morphometric analysis support the conclusion that there is a high degree of morphological and genetic diversity among *A. cerana* in China (Liu et al., 2017; Luo & Chen, 2015; Zhou et al., 2016). The completion of the *A. cerana* genome has paved.

The way for whole‐genome comparisons and implementation of SNP analysis among populations of this species (Diao et al., 2018; Park et al., 2015). One population genomics study demonstrated considerable genetic variation among populations and concluded that physical barriers were the primary driving force for the divergence of *A. cerana* (Chen et al., 2018). The authors demonstrated five distinct genetic clusters (Hainan, Tibet, Aba, Dian, and North China) (Chen et al., 2018), corresponding closely to clusters identified previously based on morphological analyses (Ge et al., 2011). The evolutionary expansion of the Asian honeybee into China and the genetic relationships among these populations are still unresolved.

Population genomics of *A.cerana* also facilitate the acquisition of data for the identification of genes involved in the adaptative evolution of *A. cerana*. A study comparing 60 eastern honeybee samples from differing altitudes in Yunan Province identified 37 genes under positive selection at higher altitudes (Montero‐Mendieta et al., 2019). Among these genes, the selection pressure on the esterase FE4‐like gene in highland *A. cerana* is thought to play a role in the regulation of metabolic activity at lower temperatures. This might contribute to earlier mating, shorter copulation, and the production of more offspring (Gilbert & Richmond, 1982). The selection pressure on other genes such as the leucokinin and NMDA receptors was hypothesized to mediate foraging in adaptation to highland habitats (Montero‐Mendieta et al., 2019). Another recent study analyzed the diversity of populations from northern and central China using the 2b‐RAD simplified genome sequencing method. The *A.cerana* samples from thirty‐one populations revealed 20 significantly enriched GO categories under selection between populations from northern and central China. The authors reported that the most significant factors were response to stimulus, signal transduction, and locomotion, indicating the importance of signal processing and response as bees adapt to different environments (Li et al., 2019).

Currently, the number of genetic groups associated with morphological clusters of *A.cerana* in China is still not resolved. The genetic relationships, geographic proximity, and environmental factors affecting these populations are not clear. Continued research on the population dynamics and evolution of *A.cerana* is needed to understand the origins and spread of the eastern honeybee and the adaptive strategies used in occupying the wide diversity of habitats in China. In this study, we performed high‐throughput DNA sequencing of representative samples of worker honeybees from diverse habitats in China and compared our results to those for the nine ecological types from a previous study (Ge et al., 2011). We included twelve resequencing published genomes of *A.cerana* collected from other regions (11 samples from Japan, 1 sample from Thailand) in our data set for comparative purposes. In addition, we examined the genetic relationships associated with geographic proximity and propose a location for the evolutionary origin of *A. cerana* in China. We also compare two populations from very different physical and climatological regions to identify differences in genetic composition and gene expression to explore the molecular basis of adaptation to environmental stress.

## 2 MATERIALS AND METHODS

### 2.1 Sample collection and DNA isolation

Samples of 138 *A. cerana* were field or colony collected from a wide variety of habitats throughout their known range in China and preserved in 75% ethanol. Forager bees were collected by sweep netting and bees from managed or wild colonies were collected with tweezers. The geographic distribution of all samples is shown in Table S1. Genomic DNA was extracted from one adult honeybee from each colony using the CTAB (cetyltrimethylammonium bromide) method and stored at 4℃ To avoid contamination from intestinal microorganisms, only the brain and thoracic muscles from adult honeybees were used.

### 2.2 Genome Sequencing and SNP Genotyping

The genomic DNA of each sample was fragmented ultrasonically, and 350‐bp length fragments were recovered from an agarose gel. The DNA was sequenced using the Illumina HiSeq sequencing platform according to the manufacturers’ instructions. The raw data are summarized in Table S2. After removing the adaptors, low‐quality (Q < 20) sequences and those reads containing more than 5% unreadable bases (N bases) were removed using the FASTX‐Toolkit software( http://hannonlab.cshl.edu/fastx_toolkit/). The resulting data are shown in Table S3. Using the alignment tool BWA (Li & Durbin, 2010), the data were mapped to an *A. cerana* reference genome (Diao et al., 2018). Reads, mapping rate, sequencing coverage, and effective depth for all 138 samples are summarized in Table S4. SNP calling was performed using GATK V4.0 (McKenna et al., 2010). Local realignment was performed to enhance the accuracy of alignments in the vicinity of indel polymorphisms. SNPs were identified and filtered using the program package of HaplotypeCaller and VariantFiltration. The parameter condition of VariantFiltration was set as "QD < 2.0 || FS > 60.0 || MQ < 40.0 || MQRankSum < −12.5 || ReadPosRankSum < −8.0 || SOR > 3.0"; 3), the SNP candidates were filtered by VCFtools (Danecek et al., 2011), and those with a minor allele frequency greater than or equal 0.05 were retained. The number of alleles is 2, and the proportion of missing data is 0.5. The sequence data are available in the NCBI database, BioProject ID: PRJNA488853. In addition, the genomic data of eleven samples from Japan and one sample from Thailand were downloaded from the public GenBank database (accession numbers SRX457260‐SRX457269, PRJDB5799, and SRX339508).

### 2.3 Population and phylogenomic analyses

Population genetic structure and individual ancestry admixture were inferred by the maximum‐likelihood routine using the expectation–maximization algorithm in the program ADMIXTURE v1.2 (Alexander et al., 2009). The predefined genetic clusters were increased from *K* = 2 to *K* = 14. Principal component analysis (PCA) of the SNPs was performed using the package EIGENSOFT (Patterson et al., 2006), and the significance level of the eigenvectors was determined using the Tracy–Widom test. The population genetic values were calculated using VCFtools (Danecek et al., 2011), including pairwise nucleotide variation as a measure of variability (*θ_π_*) and genetic differentiation (*F*_ST_). The distribution of *θ_π_* and *F*st throughout the entire genome was presented under 10‐kb window size sliding in 1‐kb steps. The number of unique and common SNPs among groups was compiled and then visualized using R language with the UpSetR package.

A neighbor‐joining phylogenetic tree was constructed using the TreeBest (Vilella et al., 2009) software with the p‐distances model. The western honeybee, *Apis mellifera*, was chosen as the outgroup and its genomic data were downloaded from the NCBI database (accession no. PRJNA236426). The bootstrap support was evaluated based on 1,000 replicates.

### 2.4 Identification of genes under selection

A comparative analysis comparing the WSichPl and Diannan populations was performed. To detect those regions showing significant selective sweep values, we examined the distribution of the *θ_π_* ratios (*θ_π_*, population1/*θ_π_*, population2) and the *F*_ST_ values. We selected windows with significantly low and high *θ_π_* ratios (the 5% left and right tails) and significantly high *F*_ST_ values (the 5% right tail) as indications of strong selective sweep along the genome. We employed XP‐EHH analysis as a second method to identify loci under selection (Danecek et al., 2011; Szpiech & Hernandez, 2014). The software package SHAPEIT was employed to phase the genotype in each chromosome and further split the haplotype file to each group of samples (Delaneau et al., 2011). Using a recombination rate of 17.4 cM/Mbp (Shi et al., 2013), we calculated the XP‐EHH value between the two groups using the selscan software tool (Szpiech & Hernandez, 2014). The top 1% of all XP‐EHH values between WSichPl and Diannan were considered as the selection signal. Only genes showing selection supported by all three methods are reported in this study (Figure 3c). Cluster of Orthologous Groups of proteins (COG) annotation for the candidate genes under selection was performed as follows: (a) Gene translations were scanned through the COG database for matches with an E‐value threshold of 1E‐10; (b) the top hit of each query sequence was considered as its annotation function; referring to the functional categories of COG annotation, the number of genes involved in the various biological process was summarized and presented (Figure 3d). In addition, the pathway enrichment analysis for the selected genes was determined using the online KOBAS tool (Xie et al., 2011). We selected *Drosophila melanogaster* as the background species. The Benjamini–Hochberg method was used for FDR correction. The FASTA protein sequence files were preprocessed for BLAST with the default cutoffs (E‐value < 10^–5^ and rank ≤ 5).

### 2.5 Transcriptome data analysis

The raw RNA‐seq data of *A. cerana* which had been subjected to treatments of 0°C and 25°C were obtained from a previous study (Xu et al., 2017). As described in that study, after extraction of the total RNAs from the whole body of bees, the three 0°C RNA libraries (ZOT‐1, ZOT‐2, and ZOT‐3) and three 25°C RNA libraries (ZRT‐1, ZRT‐2, and ZRT‐3) generated 41.56–59.70 million (M) raw reads per sample(Xu et al., 2017). The trinity software package (Grabherr et al., 2011) was used to estimate the expression levels of all genes including APICC_05210 (also called *AcVIAAT*) at 0°C and 25°C using the bowtie2, RSEM, and edgeR programs. The RNA‐seq segments were identified by matching with annotated genes of *A. cerana* using Bowtie2 (Langmead & Salzberg, 2012), and transcript quantification was determined with RSEM (Li & Dewey, 2011). Differential expression analysis of complex RNA‐seq between the two temperature conditions was performed by using edgeR (Chen et al., 2014), and the significance threshold for differential expression was set at correct *p*‐value < .05 and log_2_ (fold‐change) >2. The results were visualized as a volcano plot by R language with ggplot2.

### 2.6 Quantitative Real‐time PCR

*A.cerana* colonies were collected from the Daba mountains （location: N32°06′39.33″, E108°28′23.23″) and maintained in our apiary. Four groups of adult foraging bees (*n* = 30 bees/group) from the same hive were held in incubators for 2 hr at 0°C, 4°C, 10°C, and 25°C. Total brain RNAs from each bee were extracted using Trizol reagent and then reverse‐transcribed into cDNA to use as a PCR template using the Transcriptor First Strand cDNA Synthesis Kit (Roche). To amplify messages for the *AcVIAAT* gene, positive (5’'CGCATATCTGTTGTTACACTG'3) and negative strand (5'GACTTGAGGAAACCGAGGG'3) primers were designed for use in qRT‐PCR using the Primer premier 5.0 software. Primers for an *actin* gene (5' TGCCAACACTGTCCTTTCTG'3, 5' AGAATTGACCCACCAATCCA'3) of *A. cerana* were used as a positive control. The qPCR amplification procedure was as follows: 95°C for 30 s, followed by 40 cycles of 95°C for 5 s and 60°C for 30 s. The transcript abundance was calculated relative to the gene encoding *actin* using the 2^−ΔΔCt^ method. 25°C was used as the calibration temperature. Three biological replicates were conducted in this experiment. The *t* test was used to determine the significance of a difference among different temperature conditions.

### 2.7 Generation of *CNAG_01904*Δ and *AcVIAAT* complementation

A BLASTP search of the NCBI database indicated that the *AcVIAAT* is homologous to the *CNAG_01904 *gene of the fungus *Cryptococcus neoformans*. To test whether *AcVIAAT *functions as a* *homolog* *of the CNAG_01904 gene under conditions of abiotic stress, we knocked out the *CNAG_01904* gene in the *C. neoformans *H99 wild‐type strain using the split‐marker recombination transformation method previously described (Kim et al., 2015). To accomplish this, we first used PCR to amplify a 1‐kb‐length fragment upstream of the *CNAG_01904* locus and another 1‐kb‐length fragment downstream of the same locus using genomic DNA of *C. neoformans *H99 wild strain as templates. Two pairs of primers, TL1043/TL1044 and TL1045/TL1046 were used. Next, the resistant NEO gene was amplified by PCR using the pJAF1 plasmid as the template with the TL17/TL18 primer pair. The overlap PCR products were ligated to obtain the linear DNA composed of the upstream fragment + NEO+downstream fragment. Finally, gene gun bombardment was used to transfer the PCR product into the *C. neoformans* H99 wild‐type strain. The stable transformants were screened with diagnostic PCR using positive primers TL1049 and TL59 and negative primers TL1047/1048. Using this method, we obtained a CNAG_01904 deficient mutant of the *C. neoformans* H99 wild strain referred to here as CNAG_01904Δ.

The APICC_05210 gene was then amplified with primers TL1075/1076 using *A. cerana* cDNA as a template and cloned into the vector pTBL153 which contains the *Actin* promoter and NAT selective marker gene generating the complementation plasmid pTBL169. The SalI linearized pTBL169 was purified and biolistically transformed into CNAG_01904Δ mutant strains to generate CNAG_01904Δ::APICC_05210 strains. All information regarding the primers used in this study is shown in Table S5.

To compare the growth of our knockout, replacement, and wild‐type strains, stress sensitivity assays were performed under ten different growth conditions. These included YPD + 10°C, YPD + 20°C, YPD + 30°C, YPD + 1.5 M Sorbitol, YPD + 2.5M H_2_O_2_, YPD + 1M NaNO_2_, YPD + 1.5M KCl, YPD + 1.5M NaCl, YPD + 0.025% SDS, and YPD + Congo red. Growth on YPD at 30°C is our control growth condition. H_2_O_2_ was used to test the response to oxidative stress; SDS and Congo red were used to assay the role of the APICC_05210 gene product in the maintenance of membrane integrity; NaNO_2_ was used to test the response to nitrative stress; sorbitol was used to test the response to osmotic stress; and KCl and NaCl were used to test the response to stress from salt ions.

## 3 RESULTS

### 3.1 Population structure

**FIGURE 1:**
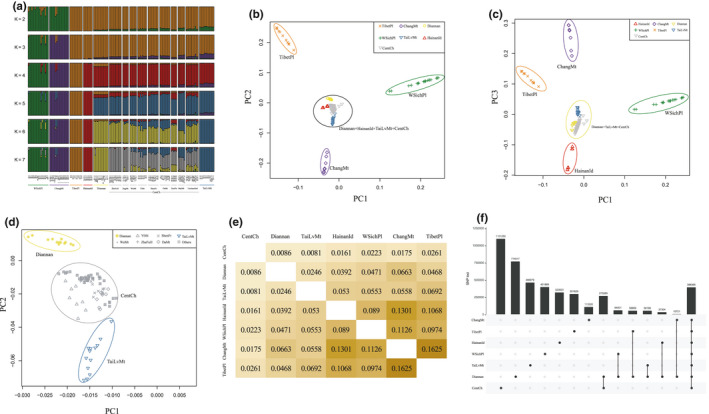
The population variation of all the surveyed samples. (a) Population admixture results showing the formation of seven populations (WSichPl, ChangMt, TibetPl, HainanId, Diannan, TaiLvMt, and CentCh). The samples from Japan and Thailand are indicated as asterisk and rectangle, respectively. (b) Four populations as determined by comparing PC1 and PC2. (c) Five populations are determined by comparing PC1 and PC3. (d) Diannan, TaiLvMt, and CentCh groups are further resolved by comparing PC1 and PC2. (e) Pairwise Fst values of the 7 populations. (f) The number of common and unique SNPs among each group. The number of SNPs shared between the Diannan group and each of the other six groups are also shown

The genome sequences of the 138 *A. cerana* samples produced 334.4 GB of raw data and resulted in 323.5GB of high‐quality sequence data with a genome coverage of 1,464X. We identified 9.17 million SNPs in the *A. cerana* genome (Table S6)*;* a number comparable to that found in *A.mellifera* (8.3 million) (Chen et al., 2018). The majority of the SNPs (68.5%) are located in intergenic regions, 27.2% are located in introns, and 4.3% are found in coding regions. STRUCTURE analysis of the 138 Chinese honeybees combined with the eleven samples from Japan and one sample from Thailand is shown in Figure 1a. When *K* = 5, the results show five clusters and one large undefined group. When *K* = 6, the TaiLvMt cluster is defined, and when *K* = 7, the Diannan population emerges (Figure 1a). STRUCTURE analysis of our collection of *A. cerana* forms seven distinct groups: those from the Western Sichuan Plateau, Chang Mountain, the Tibet Plateau, Hainan Island, Yunnan Provence, Central China and from the Taihang and Luliang Mountains. Principal component analysis (PCA) initially revealed four clusters separated in PC1‐PC2 space and five clusters separated in PC1‐PC3 space (Figure 1b and c). Further analysis in PC1‐PC2 space separates the central component into 3 populations for a total of 7 groups (Figure 1d) establishing the same 7 groups as with the STRUCTURE analysis.

Pairwise *F*st values among all the groups vary from 0.0086 to 0.1625 (Figure 1e). The highest population differentiation is between the Tibetan plateau and the northernmost Chang mountain group, which are also quite distant geographically. These two populations are the most differentiated from the other 5 populations. The CentCH population shows the lowest pairwise differentiation from the other populations (Diannan, TaiLvMt, HainanId, WSichPl, ChangMt, and TibetPl). The Central China population has the lowest pairwise FST values compared to the other 6 populations. This implies an intermixing perhaps due to the location of the central China population. The Fst values among the five subgroups (DaMt, ZheFuH, WuMt, YiMt, and ShenFr) from within the central China population are between 0.001 and 0.008 reflecting their similar genetic makeup. This is to be expected as these groups were part of the same cluster in our initial Principal component analysis implies that the topography of central China poses less geographic separation and therefore greater gene flow among the populations of central China. The maximum number of SNPs was found in the central China populations, and the minimum number was found in the collections from the ChangMt group (Figure 1f). The number of shared SNPs between the Dianan and CentCh group is significantly greater than that between the Dianan population and any of the five populations (Figure 1f). The number of common SNPs in all combinations among seven populations is shown in Figure S1.

### 3.2 Phylogenetic relationships among the populations

**FIGURE 2:**
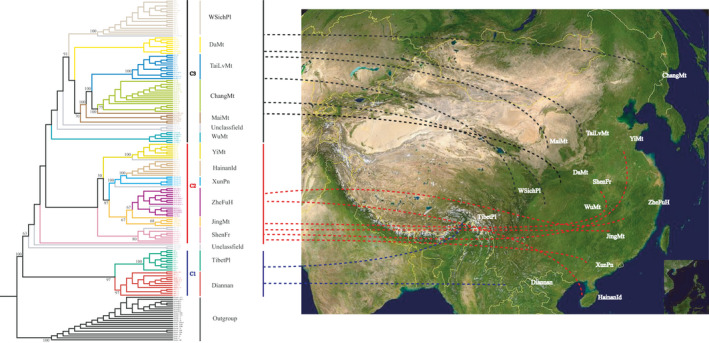
The phylogenetic relationships and geographic location of the honeybees collected. The phylogenetic relationships inferred from the SNP data. Taxa are shown as three main clades divided into localities. The map of China showing the geographic location corresponding to the group names: WSichPl (Western Sichuan Plateau); DaMt (Daba Mountain); TaiLvMt (Taihang and Luliang Mountain); ChangMt (Changbai Mountain); MaiMt (Maiji Mountain); WuMt (Wuling Mountain); YiMt (Yimeng Mountain); HainanId (Hainan Island); XunPn(Guangxi Xunyu Plain); ZheFuH(Zhejiang and Fujian hills); JingMt(Jinggangshan Mountain); ShenFr(Shennongjia Forest Area); TibetPl(Tibetan Plateau); Diannan(South Yunnan). The names of the central China population are printed in yellow

Using *A.mellifera* as the outgroup, a neighbor‐joining tree was constructed to show the phylogenetic relationships and collection sites of the 7 distinct populations from throughout China (Figure 2). The *A. cerana* populations can be assembled into three main phylogenetic groups designated C1, C2, and C3 (Figure 2). The C1 clade is composed of the semitropical Diannan and Tibet Plateau populations. Both of these populations are from Southwest China and form a sister taxon to the remaining *A. cerana* populations. The Himalayan mountains form a significant geographic barrier to genetic exchange between these two populations resulting in significant genetic differentiation between the two populations in this sister taxa.

Clade C2 is comprised of populations from Central and Eastern China and extends to Hainan Island in Southeastern China. Five of the populations, YiMt, HainanId, XunPn, ZheFuH, and JingMt (Figure 2) cluster with 97% bootstrap values with 2 subgroupings showing bootstrap values of 100% and 67%. Based on Structure Analysis, the HainanId collection forms a distinct population. Clade C3 is comprised of populations in the mountain regions ranging from southwest to northeast of China. Five of the populations (WSichPl, DaMt, TaiLvMt, ChangMt, and MaiMt) form a clade with a 91% bootstrap value. The analysis reveals relationships within this clade with 100% and 70% bootstrap values. The TaiLvMt, MaiMt, and ChangMt populations form a clade with a 100% bootstrap value. In contrast, four of the members of the central China group (DaMt, WuMt, YiMt, and TaiLvMt) show very low or no bootstrap values in Figure 2, and therefore, their phylogenetic relationships are ambiguous.

### 3.3 Genes under selection associated with the West Sichuan Plateau

Because of the extreme differences in their physical environment, we chose the WSichPI and Dianna groups to examine the molecular basis of local adaptation. The WSichPI group (West Sichuan Plateau) lies at an altitude of 3,000 meters at the junction of the southwest edge of the Qinghai–Tibet Plateau at the northern end of the Himalaya–Hengduan Mountains and the Sichuan northwest alpine canyon. The yearly average temperature is 8–9°C, and the annual precipitation is 753 mm with an average humidity of 63%. In contrast, the Dianna region in the Yunnan province is a semitropical region that lies at 500–800 meters above sea level and has an average temperature of 18–20°C with an annual precipitation of 1100–2,500 mm and an average of 82% humidity.

**FIGURE 3:**
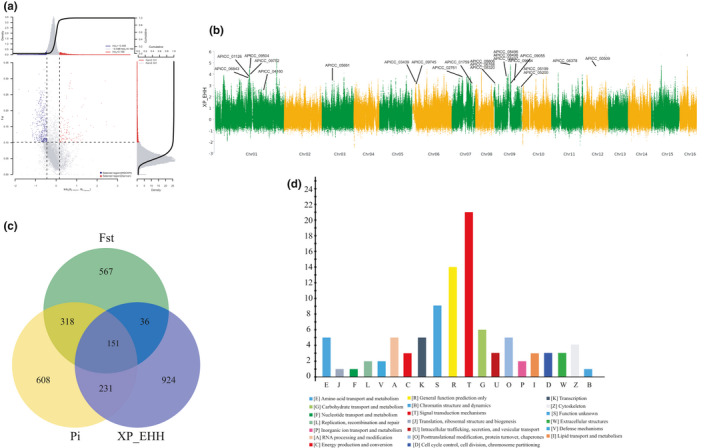
Complete identification of selection signatures for the population in a highland environment. (a) The distribution of *θ_π_* ratios (*θ_π_*_WSichPI_/*θ_π_*_Diannan_) and *F*st values. Calculation of value in 100‐kb windows sliding in 20‐kb steps. (b) The Manhattan plot of XP_EHH P‐value of SNP throughout the genomes; each point represents one SNP and the representative genes are labeled. (c) The common genes under selection comparing WSichPI and Diannan populations using the methods described above. (d) Biological process COG terms associated genes under selection in the WSichPI population

The genomic regions with selected signals for the WSichPI group were screened using two methods. The first method implemented a combination of *θ_π_* and *F*_ST_ statistics and shows regions of the genome differentially selected for the WSichPl and Diannan regions (Figure 3a). The second method was based on XP_EHH analysis (Szpiech & Hernandez, 2014; Yang et al., 2016) (Figure 3b). Finally, one hundred and fifty‐one genes were identified as being differentially selected between the WSichPl and Diannan populations (Figure 3c). Functional categories of COG (Cluster of Orthologous Groups of proteins) for these 151 candidate genes were performed. Many of these genes are involved in the COG categories of signal transduction mechanisms, carbohydrate transport and metabolism, and amino acid transport and metabolism (Figure 3d, Table S7). Three genes under selection, alpha trehalose‐phosphate synthase (*TPS*, APICC_00264), lethal (2) essential for life (*I(2)efl*,APICC_00695), and glycerol kinase (*GK*,APICC_04451), stimulate transcriptional activity in response to changes in temperature (Table S7). In the Asian lady beetle *Harmonia axyridis*, the *TPS* gene up‐regulates the level of expression under short‐time cold induction, which results in the balancing of blood sugar levels (Qin, 2012). It has been reported that the expressed level of the *TPS* gene is also up‐regulated significantly in *A. cerana* under low‐temperature conditions resulting in an increase in the total antioxidant capacity (Xia et al., 2019). The *I(2)efl* gene is a heat‐shock protein known to respond to oxidative and temperature stress in some organisms (Diao et al., 2018; Muthusamy et al., 2017; Rhee et al., 2011). Transcriptional activity of *I(2)efl* can be induced in *A. cerana* by exposure to cold temperatures (Xia et al., 2019). One of the glycerol kinase genes in the diamondback moth, *Plutella xylostella*, is known to be responsible for rapid cold hardiness associated with glycerol accumulation (Park & Kim, 2014).

Further enrichment analysis of 151 candidate genes in the KEGG pathway identifies twelve pathways associated with signal transduction or carbohydrate metabolism, two of which show significance after FDR correction (Table S8). One of the enriched KEGG pathways is the Hippo signaling pathway, considered to be important for the adaption to cold climates not only in *A. mellifera* but also in *A. cerana* (Chen et al., 2016; Chen et al., 2018).

**FIGURE 4:**
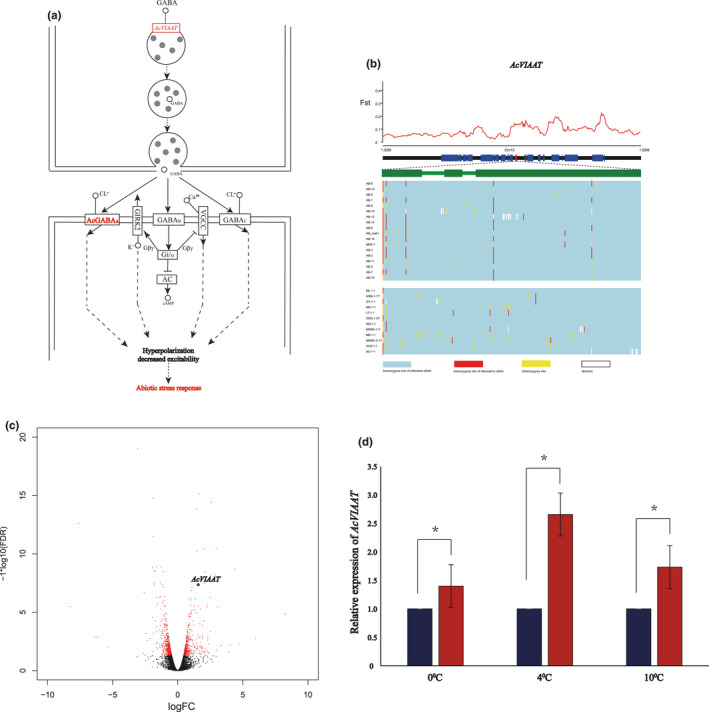
Positively selected *AcVIAAT* gene is association with cold stress. (a) A diagram illustrating the role of *AcVIAAT* in transporting GABA. (b) The selection signatures for the candidate gene. The *F*_ST_ between the WSichPI and Diannan populations is shown along the genomic regions covering the candidate genes. Red rectangles represent the candidate gene whose exons (green rectangles) are shown underneath. Below the model of the candidate gene are the SNP allele distributions for each group of *A. cerana* (yellow, heterozygous site; blue, homozygous site of reference allele; red, homozygous site of alternative allele). (c) Relative expression levels of *AcVIAAT* in *A. cerana* is up‐regulated at 0°C vs. 25°C based on transcriptome analysis. The x‐axis represents the log_2_(fold change), and the y‐axis is the ‐log_10_(FDR). Red dots represent a significant difference (FDR < 0.05). Black dots represent no significant difference. (d) qRT‐PCR of the *AcVIAAT* gene confirms the gene expression differences in *A. cerana* under low‐temperature conditions. Blue bars represent the control sample, at 25^°^C. Red bars represent the treated samples in the three low‐temperature conditions. Three biological replicates were performed for each experimental condition. *significance threshold *p* < .05

Three genes involved in the vesicular storage of gamma‐aminobutyrate acid (GABA) were determined to be under selection pressure (Figure 4a and b). Two of the genes (APICC_08806 and APICC_06768), collectively known as *AcGABA_A_*, code for a GABA alpha transporter receptor. The third gene codes for a vesicular inhibitory amino acid transporter (labeled here as *AcVIAAT*) thought to be an inhibitor of GABA uptake. Since GABA is known to be a free amino acid that helps to counteract biotic and abiotic stress in some organisms (Juge et al., 2009; Kumar et al., 2019), we further examined whether the *AcVIAAT* gene of *A. cerana* plays a role in abiotic stresses.

### 3.4 The expression of *AcVIAAT in A. cerana* at low temperature

We utilized the high‐throughput transcriptome data provided by Jiang (Xu et al., 2017) to quantify the expression of *AcVIAAT* at various temperatures. Based on the analysis of these data, the expressed level of *Ac* *VIAAT* in *A. cerana* increases significantly at 0°C compared to the 25°C control (Figure 4c), suggesting that expression level of the *Ac* *VIAAT* gene is up‐regulated at low temperature. We further measured *Ac* *VIAAT* expression in *A. cerana* at 0°C, 4°C, 10°C, and 25°C using qRT‐PCR (Figure 4d). The results show that transcriptional activity of *Ac* *VIAAT* is greater at the three lower temperatures than at 25°C. The relative level of expression is highest at 4°C. Our results indicate that *AcVIAAT* gene expression can increase in response to cold stress.

### 3.5 Replacement of *CNAG_01940* knockout with *AcVIAAT*

**FIGURE 5:**
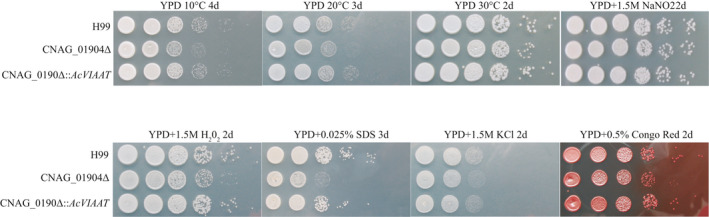
The *Ac* *VIAAT* gene is required for abiotic stress and cell membrane integrity. The *Cryptococcus* strains are indicated on the left and the conditions are indicated at the top of each panel. Cultures were grown overnight in YPD and diluted to an optical density at 600 nm of 2.0. Six tenfold serial dilutions were made in ddH_2_O, and 5 μl of each was plated on YPD and YPD with different stresses. The plates were grown at the indicated temperatures and times

To determine whether the *AcVIAAT* gene plays a role in different abiotic stresses, we used the *Cryptococcous neoformans* transformation system. The *Cryptococcus neoformans CNAG_01904* gene is homologous to the *AcVIAAT* gene in *A. cerana*. The results of our experiment show that the *AcVIAAT* gene isolated from *A. cerana* was able to complement the *Cryptococcus neoformans CNAG_01904* gene. Our comparison of the growth of the *Cryptococcus neoformans* H99 wild‐type strain, the CNAG_01904Δ (knockout strain), and the CNAG_01904Δ::APICC_05210 strain (CNAG_01904Δ restored with the *AcVIAAT*) from *A. cerana* is shown in Figure 5. Figure 5 shows that when *Cryptococcus neoformans* was grown in 30°C on YPD medium, there was a slight difference in the H99, CNAG_01904Δ and CNAG_01904Δ::APICC_05210 growth rates (Figure 5). When *Cryptococcus neoformans* is grown at 10°C or 20 °C on YPD medium, CNAG_01904Δ shows a significant decrease in growth rate compared to the H99 wild‐type and the CNAG_01904Δ::APICC_05210 rescued strains (Figure 5). Both the *Cryptococcus neoformans* H99 wild type and the *CNAG_01904*Δ::*AcVIAAT* rescue strains grew more rapidly in YPD + H_2_O_2_, YPD + SDS, YPD + KCl, and YPD + Congo red agar plates than did the CNAG_01904Δ knockout strain (Figure 5). Growth of *C. neoformans* in 1.5M NaNO_2_ was the same in all three strains (Figure 5). These results suggest that the *AcVIAAT* gene is important for maintaining cell membrane integrity and survival under oxidative stress.

## 4 DISCUSSION

The eastern honeybee *A. cerana* is thought to comprise nine ecological types in China based on morphological data and geographic location (Ge et al., 2011). Genomic analysis of populations can provide more detailed information regarding the diversity of *A. cerana* in China. A previous study based on population genomics suggested that the divergence of populations of Chinese *A. cerana* has been influenced primarily by physical–geographical factors (Chen et al., 2018). This study clarifies the evolutionary relationships among the main genetic groups and their geographical distribution. The structure and principal component analyses in Figure 1a–d show seven populations from different habitats and geographic regions. The seven genetic groups identified in this study correspond to seven of the nine ecological types described by Ge et al., 2011, suggesting a direct relationship between morphological and genetic diversity. Two ecotypes, Yun‐Gui plateau and Southern China, were not collected in this study. Previous genetic analysis did not differentiate the North China from the Changbaishan ecological types (Chen et al., 2018). We were able to separate the Changbaishan ecotype (ChangMt) when *K* = 3 and the North China ecotype (TaiLvMt) when *K* = 6 (Figure 1a). Based on our genetic and morphological analyses there was no geographic isolation among the Zhejiang hills, Fujian hills (ZheFuH), and Jinggang mountain (JingMt) samples, which are part of the CentCh population. Fixation index (Fst) is a measure of variation between populations. The low Fst values between the Diannan population from southern tropical China and the other populations analyzed (Figure 1e) suggest that either the Diannan group has a common origin with the other populations collected or there is mixing between the southern and central China populations.

Phylogenetic construction (Figure 2) shows two main clades, one composed of the combined Tibet and Diannan groups (C1) and one composed of the other bees collected. The remainder of the collection contains two clades, one from central and eastern China (C2), and one of mostly “Mountain” bees from Western and northern China (C3). The bootstrap values are low for several of the subpopulations presented. STRUCTURE, principal component, and FST analyses show a clear central China population consisting of DaMt, ZheFuH, WuMt, YiMt, and ShenFr groups (Figure 2). While the Diannan and Tibet groups form a sister taxon to the bees from the remaining locations, they are not closely related based on genetic distance (Figure 1e). We infer from this that due to their isolation of the Himalayas–Hengduan mountains and very different habitat, the Tibetan bees have a higher level of genetic difference from all other bees in this study as shown by the FST analysis.

Both the population and phylogenetic analyses are consistent with the idea that the eastern honeybee spread from tropical Asia into central China near ShenFr, resulting in a central China population consisting of ZheFuH, JingMt, DaMt, WuMt, YiMt, and ShenFr. From there some of the central China population may have moved south, forming the JingMt, XunPn, and HinanId populations. A second group moved from central China into the more mountainous regions and migrated into the WsichPl and north forming the MaiMt, TaiLvMt, and ChangMt populations. We find high bootstrap values within these two groups. The MaiMt, TaiLvMt, and ChangMt in the North form a clade with a 100% bootstrap value and 5 of the mountain populations (MaiMt, TaiLvMt, ChangMt, DaMt, and WSichPl) form a clade with a 91% bootstrap value. The central C2 group where the HainanID, XunPn, ZheFuH, and JingMt populations form a clade with a 97% bootstrap value (Figure 2).

The possible origin of *A.mellifera* has been discussed extensively (Han et al., 2012; Sheppard & Meixner, 2003; Wallberg et al., 2014; Whitfield et al., 2006). While a more recent study supports an origin for *A. mellifera* in the Middle East or North Eastern Africa (Cridland et al., 2017), fewer studies address the origin of *A.cerana*, a close relative to *A. mellifera* thought to have diverged from *A. melifera* between 6 and 25 million years ago (Arias & Sheppard, 1996). Based on our results, the geographic origin of *A.cerana* in China is the southern tropics. Our results along with the evidence that tropical Asia is a center of honeybee diversity support the origin of *A. cerana* in the tropics of Asia. Additional samples of *A. cerana* from diverse areas are needed in order to determine the origin of this species. *A.cerana* has a wide distribution in all of the countries along China's southern border. Determining the evolutionary history and expansion of *A. cerana* over its entire range is a work in progress. Whether the honeybees from Myanmar or Vietnam belong to the Dianan group, and the relationship between honeybees from the Southern Himalayas and Tibetan plateau is still not resolved. A previous study concluded that the honeybees of Hainan island, most of Indonesia, Papua New Guniea, and Sri Lanka may form a single morphocluster, based on multivariate morphometric analysis (Radloff, Hepburn, Fuchs, Otis, et al., 2005). The relationships among the *A.cerana* populations from the oceanic south Asia (Indonesia, Papua New Guniea, Malaysia, Philippines) and their relationships with the Hainan island population need to be examined and should yield interesting results.

Our genomic comparison of the Western Sichuan highland population (WSichPI) from an alpine canyon at 3,000 m and the tropical Diannan population revealed 151 genes that are subject to selection. Some of the identified genes such as *TPS*, *I(2)efl* and *GK genes* play a role in cold stress, indicative of the adaption of *A. cerana* to abiotic stress in the cold plateau environments (Park & Kim, 2014; Qin, 2012; Xia et al., 2019). The role of GABA is to inhibit neural activity thus lowering the body's response to stress. Three genes( two *AcGABA_A_* and one *Ac* *VIAAT*) in *A. cerana* involved in the GABA transport pathway are undergoing selection in the population from the western Sichuan highland. We show that *Ac* *VIAAT* (vesicular inhibitory amino acid transporter) plays a role in the response to abiotic stress including cold and oxidative stress, based on the transcriptome analysis, qPCR, and knockout experiment. To our knowledge, this is the first time the potential function of a gene related to environmental adaption has been described for *A. cerana*.

Genes undergoing positive selection related to hypoxia have been reported from a number of organisms including mammals, birds, and fish (Beall et al., 2010; Ge et al., 2013; Huerta‐Sánchez et al., 2014; Qiu et al., 2012; Qu et al., 2013; Simonson et al., 2010; Yang et al., 2015; Yi et al., 2010). In this study, we show that a gene coding for 6‐phosphofructokinase (Pfk1, APICC_02409) is under positive selection. This gene is involved in the hypoxia‐inducible transcription factors (HIFs) signaling pathway and has been shown to sense and respond to hypoxia (Azevedo et al., 2011; Gorr et al., 2006; Harrison et al., 2018). Among the 151 genes that we found to be subject to selection pressure between the two populations analyzed, some were involved in the response to environmental factors such as cold stress and hypoxia stress. However, many of the genes under selection pressure may be changing in response to biotic factors such as parasites, predators, and insect–plant interactions. Asian honeybees are a good model insect for examining the genes involved in adapting to variations in abiotic and biotic factors in the environment. Asian honeybees are a good model insect for examining the genes involved in adapting to variations in abiotic and biotic factors in the environment. The genes identified in this study as being under selective pressure will provide a valuable starting place for more detailed laboratory and field studies of *A. cerana*.

## CONFLICT OF INTEREST

The authors declare that they have no conflict of interest.

## AUTHOR CONTRIBUTION

**Peng Shi:** Data curation (lead); Investigation (lead); Writing‐original draft (equal). **Jun Zhou:** Data curation (equal); Investigation (equal). **Huali Song:** Methodology (equal); Validation (equal); Visualization (equal). **Yujuan Wu:** Methodology (equal). **Lan Lan:** Methodology (equal); Software (equal); Visualization (supporting). **Xiangyou Tang:** Formal analysis (equal); Software (equal). **Zhengang Ma:** Formal analysis (equal). **Vossbrinck R. Charles:** Validation (equal); Writing‐review & editing (equal). **Bettina Vossbrinck:** Writing‐review & editing (equal). **Zeyang Zhou:** Data curation (equal); Supervision (equal). **Jinshan Xu:** Funding acquisition (lead); Supervision (lead); Writing‐original draft (equal).

## Supporting information

Fig S1

Table S1

Table S2

Table S3

Table S4

Table S5

Table S6

Table S7

Table S8

## Data Availability

All the newly sequenced genomic data in this study are deposited in GenBank(BioProject ID: PRJNA488853) and archived in Dryad DOI (https://doi.org/10.5061/dryad.brv15dv81).
