# Supplementary figures and images for: Genomic analysis of Asian honeybee populations in China reveals evolutionary relationships and adaptation to abiotic stress
Source: Ecol Evol. 2020 Nov 2;10(23):13427–38. doi: 10.1002/ece3.6946 (PMC7713975; doi:10.1002/ece3.6946)

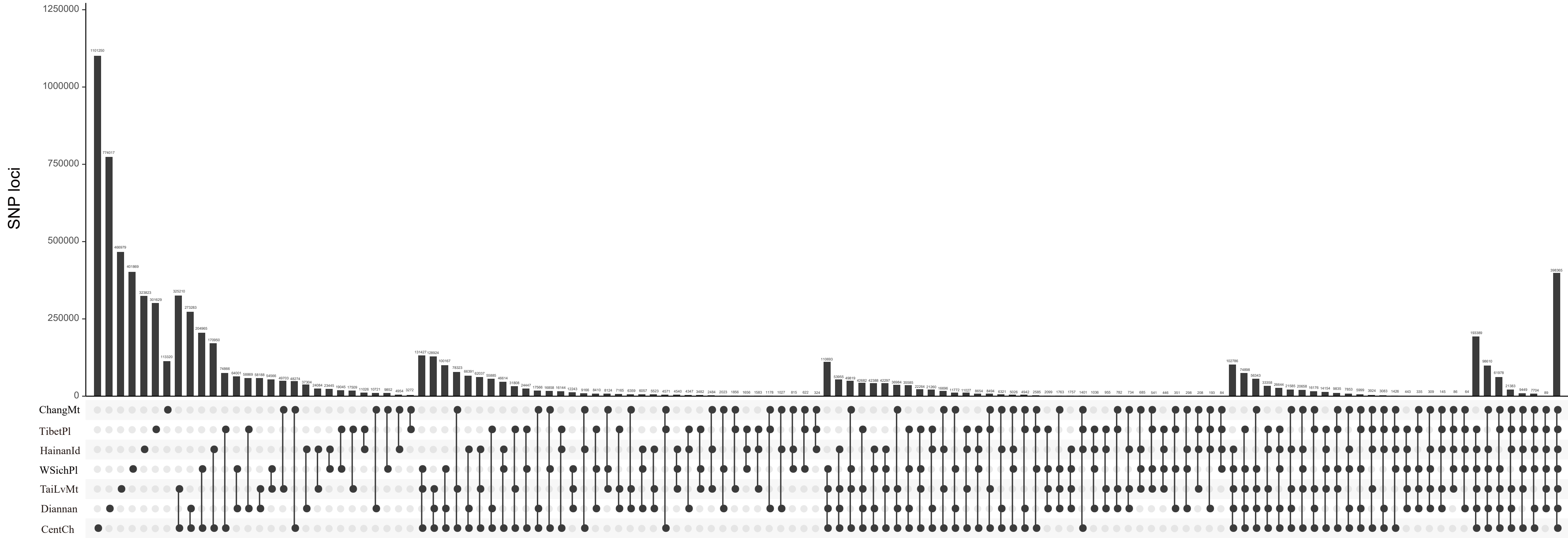

Supplement: Supplementary file 1 — Fig S1 [file ECE3-10-13427-s001.pdf]
