# Supplementary material for: Genomic analysis of Asian honeybee populations in China reveals evolutionary relationships and adaptation to abiotic stress
Source: Ecol Evol. 2020 Nov 2;10(23):13427–38. doi: 10.1002/ece3.6946 (PMC7713975; doi:10.1002/ece3.6946)
Supplement: Supplementary file 2 — Table S1 [file ECE3-10-13427-s002.docx]

Table S1. The information of geographic distribution for all collected samples in this study.

| **Samples** | [**Latitude**](javascript:;) | **Longitude** | **Sample source** | **Groups** | **Geographical distribution** |
| --- | --- | --- | --- | --- | --- |
| AT-CBQ-1 | N42°26′31.78″ | E128°05′58.34″ | managed colonies | ChangMt | Changbai Mountain |
| AT-SJZ-1 | N42°25′16.44″ | E128°06′42.64″ | managed colonies |  |  |
| AT-SJZ-2 | N42°25′16.44″ | E128°06′42.64″ | managed colonies |  |  |
| AT-SJZ-3 | N42°25′16.44″ | E128°06′42.64″ | managed colonies |  |  |
| AC-19 | N42°20′39.28″ | E126°24′54.15″ | managed colonies |  |  |
| AC-20 | N40°15′5.01″ | E124°19′43.38″ | managed colonies |  |  |
| AB_mek1 | N31°54′28.48″ | E102°12′17.45″ | managed colonies | WSichPl | Western Sichuan Plateau |
| AB-1 | N31°54′5.90″ | E102°13′22.78″ | managed colonies |  |  |
| AB-10 | N31°54′5.90″ | E102°13′22.78″ | managed colonies |  |  |
| AB-11 | N31°54′5.90″ | E102°13′22.78″ | managed colonies |  |  |
| AB-12 | N31°54′5.90″ | E102°13′22.78″ | managed colonies |  |  |
| AB-13 | N31°54′5.90″ | E102°13′22.78″ | managed colonies |  |  |
| AB-14 | N31°54′5.90″ | E102°13′22.78″ | managed colonies |  |  |
| AB-15 | N31°54′5.90″ | E102°13′22.78″ | managed colonies |  |  |
| AB-16 | N31°54′5.90″ | E102°13′22.78″ | managed colonies |  |  |
| AB-2 | N31°54′5.90″ | E102°13′22.78″ | managed colonies |  |  |
| AB-3 | N31°54′5.90″ | E102°13′22.78″ | managed colonies |  |  |
| AB-4 | N31°54′5.90″ | E102°13′22.78″ | managed colonies |  |  |
| AB-5 | N31°54′5.90″ | E102°13′22.78″ | managed colonies |  |  |
| AB-6 | N31°54′5.90″ | E102°13′22.78″ | managed colonies |  |  |
| AB-7 | N31°54′5.90″ | E102°13′22.78″ | managed colonies |  |  |
| AB-8 | N31°54′5.90″ | E102°13′22.78″ | managed colonies |  |  |
| AB-9 | N31°54′5.90″ | E102°13′22.78″ | managed colonies |  |  |
| MEK-1 | N31°54′28.48″ | E102°12′17.45″ | managed colonies |  |  |
| B-2 | N29°48′34.66″ | E95°51′16.10″ | wild individual | TibetPl | Tibet Plateau |
| B-3 | N30°06′18.22″ | E95°04′42.80″ | wild individual |  |  |
| B-3-1 | N30°06′18.22″ | E95°04′42.80″ | wild individual |  |  |
| B-3-2 | N30°06′18.22″ | E95°04′42.80″ | wild individual |  |  |
| B-4 | N30°16′15.99″ | E94°49′1.67″ | wild individual |  |  |
| B-4-1 | N30°16′15.99″ | E94°49′1.67″ | wild individual |  |  |
| B-4-2 | N30°16′15.99″ | E94°49′1.67″ | wild individual |  |  |
| B-5 | N30°16′15.99″ | E94°49′1.67″ | wild individual |  |  |
| B-5-1 | N30°16′15.99″ | E94°49′1.67″ | wild individual |  |  |
| B-5-2 | N30°16′15.99″ | E94°49′1.67″ | wild individual |  |  |
| M-10 | N29°08′9.20″ | E93°42′9.92″ | wild individual |  |  |
| BS-LL-1 | N19°06′2.21″ | E109°25′12.47″ | managed colonies |  |  |
| BS-LZ-1 | N19°19′27.12″ | E109°27′23.38″ | managed colonies |  |  |
| BS-LZ-2 | N19°19′27.12″ | E109°27′23.38″ | managed colonies |  |  |
| BS-LZ-4 | N19°19′27.12″ | E109°27′23.38″ | managed colonies | HainanId | Hainan Island |
| BS-LZ-6 | N19°19′27.12″ | E109°27′23.38″ | managed colonies |  |  |
| TC-AD-1 | N19°21′41.69″ | E110°04′55.37″ | wild colonies |  |  |
| WN-LJ-2 | N18°47′48.13″ | E110°23′12.01″ | wild colonies |  |  |
| DA-LK-1 | N19°20′39.55″ | E110°18′14.23″ | wild colonies |  |  |
| ML-1-1 | N22°34′12.23″ | E100°34′53.26″ | wild colonies | Diannan | Diannan |
| MXB-1-1Y | N22°41′24.66″ | E100°56′26.53″ | wild colonies |  |  |
| MXNC-1-Y | N22°43′21.59″ | E100°56′10.06″ | wild colonies |  |  |
| MXNC-3-1Y | N22°43′21.59″ | E100°56′10.06″ | wild colonies |  |  |
| MZ-1-1 | N21°59′18.68″ | E100°15′58.98″ | wild colonies |  |  |
| NN-1-1 | N23°47′35.19″ | E100°16′16.71″ | wild colonies |  |  |
| NQ-1-1 | N21°26′45.29″ | E101°41′24.42″ | wild colonies |  |  |
| XHS-1-1 | N22°05′34.29″ | E100°11′31.23″ | wild colonies |  |  |
| DDG-1-2Y | N22°22′16.58″ | E100°55′52.76″ | wild colonies |  |  |
| DY-1-1 | N21°12′26.70″ | E101°42′1.64″ | wild colonies |  |  |
| LT-1-1 | N22°41′3.85″ | E100°39′47.31″ | wild colonies |  |  |
| MJ-1-1 | N23°25′53.32″ | E101°40′38.18″ | wild individual |  |  |
| WYS-DWA-1 | N28°0′18.38″ | E118°11′27.86″ | wild colonies | ZheFuH | Zhejiang and Fujian hills |
| WYS-LHF-1 | N27°41′25.43″ | E117°56′29.64″ | wild colonies |  |  |
| WYS-TZZ-1 | N27°45′35.19″ | E118°01′48.55″ | wild colonies |  |  |
| WYS-XFJL-1 | N27°38′26.51″ | E117°59′3.08″ | wild colonies |  |  |
| YX-PK-1 | N24°04′16.05″ | E117°19′53.66″ | managed colonies |  |  |
| YX-YS-2 | N23°57′25.57″ | N23°57′25.57″ | managed colonies |  |  |
| ST-KYZ-1 | N30°12′38.10″ | E117°28′40.05″ | wild colonies |  |  |
| ST-SC-1 | N30°09′4.42″ | E117°35′25.19″ | wild colonies |  |  |
| SR-FJA-1 | N28°05′58.32″ | E118°07′49.74″ | managed colonies |  |  |
| SR-FJA-2 | N28°05′58.32″ | E118°07′49.74″ | managed colonies |  |  |
| GNJ-XD-1 | N29°53′50.43″ | E117°30′25.44″ | wild colonies |  |  |
| YL-TYD-1 | N26°29′47.32″ | E114°02′43.42″ | wild colonies | JingMt | Jinggangshan |
| YL-TYD-2 | N26°30′13.22″ | N26°30′13.22″ | wild colonies |  |  |
| JGS-LJP-1 | N26°33′25.28″ | E114°10′10.45″ | wild colonies |  |  |
| JGS-LZ-1 | N26°34′13.35″ | E114°09′45.57″ | wild colonies |  |  |
| JXND-1 | N28°45′57.56″ | E115°49′40.23″ | wild colonies |  |  |
| FJS-LJB | N27°50′34.18″ | E108°46′4.13″ | wild colonies | WuMt | Wuling Mountain |
| KC-MCH-1 | N27°52′51.52″ | E108°48′11.82″ | wild colonies |  |  |
| KC-MCH-2 | N27°52′51.52″ | E108°48′11.82″ | wild colonies |  |  |
| KW-DZ | N27°54′55.39″ | E108°49′35.45″ | wild colonies |  |  |
| YY-NEY-1 | N28°42′5.86″ | E108°30′44.44″ | managed colonies |  |  |
| YY-TG-1 | N28°21′36.11″ | E108°46′51.77″ | managed colonies |  |  |
| AC-5 | N35°13′23.79″ | E117°54′13.90″ | managed colonies | YiMt | Yimeng Mountain |
| AC-6 | N35°13′23.79″ | E117°54′13.90″ | managed colonies |  |  |
| AC-13 | N35°33′34.85″ | E117°58′7.02″ | managed colonies |  |  |
| AC-14 | N35°33′34.85″ | E117°58′7.02″ | managed colonies |  |  |
| AC-15 | N35°14′52.86″ | E117°55′46.19″ | wild colonies |  |  |
| AC-16 | N35°14′58.05″ | E117°52′56.17″ | managed colonies |  |  |
| AC-17 | N36°18′48.17″ | E118°31′11.07″ | managed colonies |  |  |
| AC-18 | N36°18′48.17″ | E118°31′11.07″ | managed colonies |  |  |
| FS-TH-1 | N29°01′21.16″ | E111°29′24.29″ | managed colonies | ShenFr | Shennongjia Forest Area |
| SB-QQ-1 | N31°44′56.85″ | E110°39′23.41″ | managed colonies |  |  |
| XH-DN-1 | N31°44′44.39″ | E110°40′31.28″ | managed colonies |  |  |
| XS-LHK-1 | N31°12′48.02″ | E110°52′52.09″ | managed colonies |  |  |
| NY-HZB-1 | N31°21′53.92″ | E110°36′35.86″ | wild colonies |  |  |
| MY-SLT-2 | N31°26′16.86″ | E110°26′32.40″ | wild colonies |  |  |
| HP-HH-1 | N31°39′25.50″ | E110°28′7.85″ | managed colonies |  |  |
| HB_jm1 | N31°10′36.39″ | E112°48′56.16″ | managed colonies |  |  |
| HB_jm2 | N31°18′35.07″ | E113°03′18.48″ | managed colonies |  |  |
| AC-1 | N35°49′12.82″ | E113°19′45.44″ | wild colonies | DaMt | Daba Mountain |
| CK_bslm1 | N32°06′39.33″ | E108°28′23.23″ | wild colonies |  |  |
| CK-bs | N32°06′39.33″ | E108°28′23.23″ | wild colonies |  |  |
| CK-bslm2 | N32°06′39.33″ | E108°28′23.23″ | wild colonies |  |  |
| CK-daxl | N31°46′33.54″ | E109°05′41.16″ | wild colonies |  |  |
| CK-hab | N31°50′19.29″ | E109°05′46.89″ | wild colonies |  |  |
| CK-qq | N31°57′5.73″ | E108°39′32.55″ | wild colonies |  |  |
| SQ-1-3 | N33°07′40.26″ | E108°10′34.04″ | wild colonies |  |  |
| ZZ-1-1y | N33°44′1.63″ | E107°58′17.25″ | wild colonies |  |  |
| PN-SG-1b | N23°23′6.36″ | E110°30′38.41″ | managed colonies | XunPn | Guangxi Xunyu Plain |
| PN-SG-1y | N23°23′6.36″ | E110°30′38.41″ | managed colonies |  |  |
| PN-SG-2b | N23°23′6.36″ | E110°30′38.41″ | managed colonies |  |  |
| PN-SG-3b | N23°23′6.36″ | E110°30′38.41″ | managed colonies |  |  |
| PN-SG-3y | N23°23′6.36″ | E110°30′38.41″ | managed colonies |  |  |
| ZZ-2-1y | N33°49′2.43″ | E108°0′37.11″ | wild colonies | MaiMt | Maiji Mountain |
| FP-XMG-2 | N33°40′12.28″ | E107°58′7.47″ | wild colonies |  |  |
| MJQ-DC2-1 | N34°19′46.48″ | E106°22′27.67″ | managed colonies |  |  |
| MJQ-DL-1 | N34°20′52.64″ | E106°38′31.08″ | managed colonies |  |  |
| MJQ-TGZ2 | N34°23′22.38″ | E105°59′53.96″ | managed colonies |  |  |
| LD-LPSF-1y2 | N35°26′49.65″ | E106°11′49.94″ | wild colonies |  |  |
| SR-HX-1 | N28°39′36.12″ | E117°57′27.43″ | managed colonies | Unclassfied | Unclassfied |
| CS-JS-2 | N28°27′20.13″ | E105°57′56.31″ | wild colonies |  |  |
| CS-JSTZY | N28°27′20.53″ | E105°57′57.52″ | wild colonies |  |  |
| HB-LC-1 | N30°17′37.52″ | E108°55′55.31″ | managed colonies |  |  |
| HC-1 | N29°58′28.99″ | E106°16′20.34″ | wild individual |  |  |
| JMX-FXL2 | N28°45′43.20″ | E110°25′57.31″ | wild colonies |  |  |
| JMX-ZMK1 | N28°50′39.04″ | E110°26′47.70″ | wild colonies |  |  |
| JMX-ZS-1 | N28°45′43.20″ | E110°25′57.31″ | wild colonies |  |  |
| JY-YCZ-1ys | N35°34′58.13″ | E106°27′43.59″ | wild colonies |  |  |
| KC-DT-1 | N27°52′51.52″ | E108°48′11.82″ | wild colonies |  |  |
| KC-DT-4 | N27°52′51.52″ | E108°48′11.82″ | wild colonies |  |  |
| AC-10 | N35°43′7.95″ | E112°05′49.68″ | wild colonies | TaiLvMt | Taihang and Luliang Mountain |
| AC-11 | N35°47′44.16″ | E113°27′37.93″ | wild colonies |  |  |
| AC-12 | N35°47′44.16″ | E113°27′37.93″ | wild colonies |  |  |
| AC-2 | N31°54′5.90″ | E102°13′22.78″ | wild colonies |  |  |
| AC-3 | N31°54′5.90″ | E102°13′22.78″ | wild colonies |  |  |
| AC-4 | N31°54′5.90″ | E102°13′22.78″ | wild colonies |  |  |
| AC-7 | N31°54′5.90″ | E102°13′22.78″ | wild colonies |  |  |
| AC-8 | N31°54′5.90″ | E102°13′22.78″ | wild colonies |  |  |
| AC-9 | N31°54′5.90″ | E102°13′22.78″ | wild colonies |  |  |
| HLOLZ-1-2 | N35°35′5.43″ | E109°50′6.63″ | managed colonies |  |  |
| HLO-YFJ1 | N35°35′5.43″ | E109°50′6.63″ | managed colonies |  |  |
| HLQC-1-1 | N35°41′32.99″ | E109°10′45.93″ | managed colonies |  |  |
| JC-1-1 | N35°21′27.29″ | E107°26′38.69″ | wild individual |  |  |
