# Supplementary material for: Genomic analysis of Asian honeybee populations in China reveals evolutionary relationships and adaptation to abiotic stress
Source: Ecol Evol. 2020 Nov 2;10(23):13427–38. doi: 10.1002/ece3.6946 (PMC7713975; doi:10.1002/ece3.6946)
Supplement: Supplementary file 3 — Table S2 [file ECE3-10-13427-s003.doc]

Table S2.    The production of raw data for all the sequenced samples based on high-throughput sequencing 

Samples	Reads	Bases(nt)	Error rate(%)	Q20(%)	Q30(%)	GC(%)	
AB_mek1	18,010,928	2,701,639,200	0.39	96.88	93.11	34.19	
AB-1	12,610,608	1,891,591,200	0.49	95.06	92.49	33.35	
AB-10	14,613,292	2,191,993,800	0.49	95.10	92.56	33.40	
AB-11	19,073,002	2,860,950,300	0.29	97.58	96.30	33.70	
AB-12	13,652,978	2,047,946,700	0.69	92.67	89.49	33.16	
AB-13	13,058,344	1,958,751,600	0.49	95.14	92.61	33.50	
AB-14	17,788,114	2,668,217,100	0.30	97.50	96.17	33.98	
AB-15	12,937,544	1,940,631,600	0.53	94.69	92.06	33.40	
AB-16	15,729,974	2,359,496,100	0.52	94.73	92.07	33.12	
AB-2	13,330,216	1,999,532,400	0.52	94.83	92.22	32.99	
AB-3	13,832,166	2,074,824,900	0.49	95.14	92.61	33.17	
AB-4	15,093,004	2,263,950,600	0.51	94.95	92.36	32.92	
AB-5	15,877,320	2,381,598,000	0.49	95.10	92.53	33.15	
AB-6	16,318,346	2,447,751,900	0.29	97.53	96.21	33.80	
AB-7	17,020,154	2,553,023,100	0.42	96.30	90.35	32.55	
AB-8	19,288,964	2,893,344,600	0.44	96.05	89.92	32.74	
AB-9
	15,535,990	2,330,398,500	0.53	94.64	91.97	33.09	
AT-CBQ-1	10,125,228	3,037,568,400	0.46	95.61	93.65	34.04	
AT-SJZ-1	9,472,607	2,841,782,100	0.32	97.32	95.87	33.87	
AT-SJZ-2	8,346,212	2,503,863,600	0.30	97.52	96.19	34.14	
AT-SJZ-3	5,544,157	1,663,247,100	0.31	97.39	96.03	34.28	
B-2	10,746,920	1,612,038,000	0.29	97.57	95.17	33.16	
B-3	9,781,618	1,467,242,700	0.29	97.67	95.38	33.64	
B-3-1	13,689,272	2,053,390,800	0.53	94.72	92.08	33.52	
B-3-2	14,901,998	2,235,299,700	0.50	94.97	92.39	33.27	
B-4	10,219,008	1,532,851,200	0.29	97.66	95.36	33.69	
B-4-1	13,778,730	2,066,809,500	0.51	94.95	92.35	33.45	
B-4-2	12,891,404	1,933,710,600	0.51	94.89	92.26	33.37	
B-5	11,519,330	1,727,899,500	0.35	96.83	93.88	33.08	
B-5-1	13,869,754	2,080,463,100	0.49	95.05	92.49	33.05	
B-5-2	13,088,588	1,963,288,200	0.50	95.06	92.48	33.27	
BS-LL-1	13,977,654	2,096,648,100	0.47	95.66	89.78	32.90	
BS-LZ-1	14,174,326	2,126,148,900	0.46	95.75	89.94	33.15	
BS-LZ-2	13,590,580	2,038,587,000	0.46	95.67	89.84	32.81	
BS-LZ-4	12,772,940	1,915,941,000	0.47	95.57	89.61	33.23	
BS-LZ-6	13,600,490	2,040,073,500	0.47	95.58	89.68	33.27	
CK_bslm1	14,267,086	2,140,062,900	0.42	96.62	92.69	33.45	
CK-bs	13,050,926	1,957,638,900	0.48	95.94	92.03	33.24	
CK-bslm2	12,898,698	1,934,804,700	0.49	95.88	91.92	35.64	
CK-daxl	15,716,476	2,357,471,400	0.53	95.54	91.25	33.74	
CK-hab	16,656,476	2,498,471,400	0.53	95.61	91.25	36.66	
CK-qq	35,793,020	5,404,746,020	0.42	96.08	90.67	33.35	
CS-JS-2	18,845,870	2,826,880,500	0.59	94.32	88.19	33.72	
CS-JSTZY	16,424,154	2,463,623,100	0.79	92.25	84.59	33.67	
DA-LK-1	12,308,184	1,846,227,600	0.45	95.83	90.28	33.23	
DDG-1-2Y	10,958,158	1,643,723,700	0.53	95.48	91.22	35.64	
DY-1-1	10,704,948	1,605,742,200	0.28	97.78	95.70	33.55	
FJS-LJB	15,371,286	2,305,692,900	0.60	94.19	87.90	32.85	
FP-XMG-2	14,060,548	2,109,082,200	0.28	97.84	95.83	33.45	
FS-TH-1	13,914,286	2,087,142,900	0.28	97.78	95.74	33.12	
GNJ-XD-1	9,689,522	1,453,428,300	0.35	96.92	94.03	33.19	
HB_jm1	16,260,214	2,439,032,100	0.40	96.80	93.00	33.16	
HB_jm2	12,651,564	1,897,734,600	0.42	96.61	92.49	33.34	
HB-LC-1	16,268,126	2,440,218,900	0.65	93.69	87.09	33.33	
HC-1	14,625,660	2,193,849,000	0.32	97.31	94.64	33.38	
HLOLZ-1-2	12,101,244	1,815,186,600	0.42	96.65	92.56	33.91	
HLO-YFJ1	13,255,144	1,988,271,600	0.28	97.70	95.56	33.79	
HLQC-1-1	11,117,088	1,667,563,200	0.49	95.81	91.68	32.92	
HP-HH-1	16,970,558	2,545,583,700	0.57	94.62	88.62	33.05	
JC-1-1	12,156,306	1,823,445,900	0.48	95.89	91.71	33.31	
JGS-LJP-1	12,230,440	1,834,566,000	0.34	97.06	94.29	33.56	
JGS-LZ-1	11,023,104	1,653,465,600	0.34	97.10	94.36	33.30	
JMX-FXL2	15,653,704	2,348,055,600	0.57	94.53	88.44	32.22	
JMX-ZMK1	11,374,170	1,706,125,500	0.62	93.97	87.52	32.01	
JMX-ZS-1	13,996,822	2,099,523,300	0.57	94.55	88.52	32.00	
JXND-1	11,696,968	1,754,545,200	0.35	96.89	93.97	33.53	
JY-YCZ-1ys	9,929,800	1,489,470,000	0.51	95.72	91.44	33.19	
KC-DT-1	18,906,678	2,836,001,700	0.64	93.83	87.19	32.98	
KC-DT-4	14,021,606	2,103,240,900	0.60	94.22	87.92	33.09	
KC-MCH-1	18,267,664	2,740,149,600	0.64	93.89	87.25	32.91	
KC-MCH-2	12,420,460	1,863,069,000	0.47	95.69	89.81	33.24	
KW-DZ	13,768,000	2,065,200,000	0.47	95.61	89.67	32.98	
LD-LPSF-1y2	9,836,610	1,475,491,500	0.51	95.64	91.41	33.21	
LT-1-1	11,470,100	1,720,515,000	0.56	95.17	90.53	33.60	
M-10	12,004,484	1,800,672,600	0.29	97.68	95.39	33.05	
MEK-1	15,502,136	2,325,320,400	0.28	97.63	96.37	33.57	
MJ-1-1	10,323,742	1,548,561,300	0.55	95.30	90.83	34.19	
MJQ-DC2-1	10,097,874	1,514,681,100	0.49	95.76	91.56	33.62	
MJQ-DL-1	10,785,086	1,617,762,900	0.28	97.75	95.67	33.29	
MJQ-TGZ2	13,257,698	1,988,654,700	0.45	95.70	92.77	33.55	
ML-1-1	10,840,152	1,626,022,800	0.53	95.46	91.03	33.42	
MXB-1-1Y	36,419,458	5,499,338,158	0.42	96.06	90.65	33.70	
MXNC-1-Y	12,993,484	1,949,022,600	0.28	97.76	95.67	33.86	
MXNC-3-1Y	35,920,520	5,423,998,520	0.44	95.88	90.26	34.82	
MY-SLT-2	17,419,982	2,612,997,300	0.60	94.26	88.00	33.53	
MZ-1-1	15,473,060	2,320,959,000	0.28	97.77	95.69	34.02	
NN-1-1	9,859,772	1,478,965,800	0.58	95.01	90.33	33.40	
NQ-1-1	9,883,396	1,482,509,400	0.53	95.50	91.12	33.16	
NY-HZB-1	12,334,200	1,850,130,000	0.57	94.59	88.61	32.95	
PN-SG-1b	14,263,796	2,139,569,400	0.46	95.70	90.19	33.23	
PN-SG-1y	12,967,782	1,945,167,300	0.44	95.89	90.33	32.75	
PN-SG-2b	16,750,828	2,512,624,200	0.45	95.78	90.28	33.09	
PN-SG-3b	14,125,914	2,118,887,100	0.57	94.45	87.61	32.68	
PN-SG-3y	16,834,052	2,525,107,800	0.42	95.99	90.72	32.80	
SB-QQ-1	15,026,998	2,254,049,700	0.57	94.60	88.69	32.72	
SQ-1-3	39,986,082	6,037,898,382	0.43	96.01	90.56	34.05	
SR-FJA-1	11,117,542	1,667,631,300	0.36	96.78	93.78	33.74	
SR-FJA-2	11,226,278	1,683,941,700	0.35	96.90	93.98	33.24	
SR-HX-1	12,316,430	1,847,464,500	0.36	96.72	93.66	33.35	
ST-KYZ-1	12,687,474	1,903,121,100	0.34	97.01	94.19	33.30	
ST-SC-1	11,523,386	1,728,507,900	0.36	96.83	93.86	33.08	
TC-AD-1	13,124,610	1,968,691,500	0.45	95.83	90.24	32.66	
WN-LJ-2	14,165,470	2,124,820,500	0.45	95.78	90.10	33.05	
WYS-DWA-1	16,635,680	2,495,352,000	0.29	97.62	96.34	34.35	
WYS-LHF-1	12,092,180	1,813,827,000	0.35	96.87	93.94	33.38	
WYS-TZZ-1	11,014,952	1,652,242,800	0.41	96.12	92.27	32.46	
WYS-XFJL-1	10,797,366	1,619,604,900	0.35	96.95	94.08	32.91	
XH-DN-1	13,965,360	2,094,804,000	0.57	94.50	88.45	33.37	
XHS-1-1	11,561,610	1,734,241,500	0.53	95.50	91.14	34.13	
XS-LHK-1	12,853,122	1,927,968,300	0.58	94.47	88.42	32.08	
YL-TYD-1	9,599,068	1,439,860,200	0.35	96.94	94.08	33.54	
YL-TYD-2	12,791,224	1,918,683,600	0.34	97.09	94.36	33.88	
YX-PK-1	10,832,680	1,624,902,000	0.35	96.88	93.97	33.32	
YX-YS-2	11,616,800	1,742,520,000	0.33	97.12	94.39	33.34	
YY-NEY-1	14,801,158	2,220,173,700	0.59	94.33	88.06	32.12	
YY-TG-1	17,968,624	2,695,293,600	0.57	94.56	88.64	32.16	
ZZ-1-1y	32,202,318	4,862,550,018	0.44	95.89	90.30	33.98	
ZZ-2-1y	14,367,862	2,155,179,300	0.43	96.48	92.34	33.73	
AC-1	25,053,760	3,758,064,000	0.46	97.66	93.49	33.38	
AC-10	23,891,736	3,583,760,400	0.43	97.98	94.22	33.51	
AC-11	23,719,686	3,557,952,900	0.44	97.83	93.85	33.16	
AC-12	24,548,940	3,682,341,000	0.44	97.93	94.11	33.10	
AC-13	23,045,484	3,456,822,600	0.64	95.84	89.66	32.42	
AC-14	23,861,862	3,579,279,300	0.44	97.95	94.15	33.02	
AC-15	21,967,268	3,295,090,200	0.42	98.06	94.40	33.28	
AC-16	26,224,210	3,933,631,500	0.43	97.99	94.29	35.01	
AC-17	26,134,640	3,920,196,000	0.46	97.71	93.56	33.15	
AC-18	26,138,172	3,920,725,800	0.43	97.95	94.13	33.05	
AC-19	22,960,226	3,444,033,900	0.46	97.69	93.53	33.56	
AC-2	20,979,066	3,146,859,900	0.42	98.07	94.48	33.59	
AC-20	26,378,704	3,956,805,600	0.44	97.83	93.84	32.80	
AC-3	26,440,286	3,966,042,900	0.42	98.06	94.40	33.27	
AC-4	26,990,718	4,048,607,700	0.45	97.74	93.70	33.63	
AC-5	23,057,838	3,458,675,700	0.43	98.01	94.29	33.93	
AC-6	20,837,886	3,125,682,900	0.44	97.82	93.87	33.40	
AC-7	22,958,528	3,443,779,200	0.43	98.00	94.29	33.60	
AC-8	23,969,914	3,595,487,100	0.43	98.02	94.36	33.45	
AC-9	28,160,516	4,224,077,400	0.45	97.81	93.85	33.16	
