# Supplementary material for: Genomic analysis of Asian honeybee populations in China reveals evolutionary relationships and adaptation to abiotic stress
Source: Ecol Evol. 2020 Nov 2;10(23):13427–38. doi: 10.1002/ece3.6946 (PMC7713975; doi:10.1002/ece3.6946)
Supplement: Supplementary file 4 — Table S3 [file ECE3-10-13427-s004.rtf]

Table S3.    The production of clean data for all the sequenced samples based on high-throughput sequencing


Samples	Reads	Bases(nt)	Error rate(%)	Q20(%)	Q30(%)	GC(%)	
AB_mek1	17,661,732	2,649,259,800	0.38	97	93.24	34.03	
AB-1	12,551,194	1,882,679,100	0.49	95.11	92.53	33.32	
AB-10	14,540,208	2,181,031,200	0.49	95.13	92.59	33.36	
AB-11	18,993,242	2,848,986,300	0.28	97.62	96.34	33.66	
AB-12	13,594,562	2,039,184,300	0.69	92.7	89.52	33.12	
AB-13	12,990,168	1,948,525,200	0.48	95.18	92.65	33.46	
AB-14	17,717,916	2,657,687,400	0.29	97.53	96.21	33.95	
AB-15	12,858,622	1,928,793,300	0.52	94.73	92.09	33.35	
AB-16	15,648,064	2,347,209,600	0.52	94.75	92.11	33.09	
AB-2	13,262,054	1,989,308,100	0.51	94.86	92.27	32.95	
AB-3	13,760,924	2,064,138,600	0.48	95.18	92.64	33.13	
AB-4	15,018,024	2,252,703,600	0.5	95	92.41	32.88	
AB-5	15,802,290	2,370,343,500	0.49	95.14	92.57	33.12	
AB-6	16,253,230	2,437,984,500	0.29	97.56	96.25	33.76	
AB-7	16,933,814	2,540,072,100	0.41	96.34	90.41	32.49	
AB-8	19,180,774	2,877,116,100	0.44	96.11	89.98	32.69	
AB-9	15,453,478	2,318,021,700	0.53	94.67	92.01	33.05	
AT-CBQ-1	20,158,760	3,023,814,000	0.45	95.65	93.68	34.01	
AT-SJZ-1	18,871,712	2,830,756,800	0.32	97.35	95.91	33.84	
AT-SJZ-2	16,629,788	2,494,468,200	0.3	97.55	96.22	34.1	
AT-SJZ-3	11,047,028	1,657,054,200	0.3	97.42	96.07	34.25	
B-2	10,721,412	1,608,211,800	0.3	97.59	95.2	33.15	
B-3	9,754,130	1,463,119,500	0.28	97.69	95.42	33.6	
B-3-1	13,630,666	2,044,599,900	0.52	94.75	92.11	33.48	
B-3-2	14,838,002	2,225,700,300	0.5	95.01	92.42	33.23	
B-4	10,192,906	1,528,935,900	0.29	97.68	95.39	33.66	
B-4-1	13,718,256	2,057,738,400	0.51	94.98	92.38	33.42	
B-4-2	12,841,270	1,926,190,500	0.51	94.92	92.28	33.34	
B-5	11,491,768	1,723,765,200	0.35	96.86	93.92	33.05	
B-5-1	13,808,334	2,071,250,100	0.49	95.09	92.53	33.02	
B-5-2	13,024,592	1,953,688,800	0.49	95.09	92.52	33.23	
BS-LL-1	13,950,616	2,092,592,400	0.46	95.69	89.83	32.88	
BS-LZ-1	14,143,324	2,121,498,600	0.46	95.78	90	33.12	
BS-LZ-2	13,553,742	2,033,061,300	0.46	95.72	89.92	32.78	
BS-LZ-4	12,744,980	1,911,747,000	0.47	95.61	89.66	33.21	
BS-LZ-6	13,566,262	2,034,939,300	0.47	95.64	89.75	33.23	
CK_bslm1	13,510,916	2,026,637,400	0.41	96.78	92.8	33.14	
CK-bs	12,522,862	1,878,429,300	0.46	96.11	92.18	32.98	
CK-bslm2	12,202,728	1,830,409,200	0.47	96.06	92.05	35.32	
CK-daxl	14,483,430	2,172,514,500	0.54	95.52	91.08	33.38	
CK-hab	15,922,802	2,388,420,300	0.53	95.64	91.22	36.25	
CK-qq	34,828,272	5,259,069,072	0.42	96.12	90.7	33.23	
CS-JS-2	18,649,432	2,797,414,800	0.58	94.43	88.34	33.69	
CS-JSTZY	16,246,828	2,437,024,200	0.78	92.36	84.72	33.65	
DA-LK-1	12,281,472	1,842,220,800	0.45	95.86	90.33	33.2	
DDG-1-2Y	10,381,782	1,557,267,300	0.51	95.68	91.39	35.4	
DY-1-1	10,668,662	1,600,299,300	0.28	97.81	95.75	33.52	
FJS-LJB	14,779,066	2,216,859,900	0.58	94.45	88.21	32.83	
FP-XMG-2	14,019,830	2,102,974,500	0.27	97.86	95.86	33.42	
FS-TH-1	13,843,906	2,076,585,900	0.28	97.81	95.78	33.09	
GNJ-XD-1	9,669,140	1,450,371,000	0.34	96.95	94.06	33.16	
HB_jm1	15,592,260	2,338,839,000	0.39	96.88	93.05	32.95	
HB_jm2	12,481,576	1,872,236,400	0.41	96.69	92.56	33.23	
HB-LC-1	16,123,396	2,418,509,400	0.65	93.78	87.22	33.31	
HC-1	14,594,602	2,189,190,300	0.32	97.33	94.67	33.35	
HLOLZ-1-2	11,957,804	1,793,670,600	0.41	96.73	92.66	33.79	
HLO-YFJ1	13,220,928	1,983,139,200	0.28	97.73	95.6	33.76	
HLQC-1-1	10,665,156	1,599,773,400	0.49	95.85	91.67	32.72	
HP-HH-1	16,319,018	2,447,852,700	0.54	94.89	88.93	33.03	
JC-1-1	11,949,066	1,792,359,900	0.48	95.95	91.78	33.2	
JGS-LJP-1	12,198,702	1,829,805,300	0.34	97.08	94.32	33.53	
JGS-LZ-1	10,998,234	1,649,735,100	0.34	97.12	94.39	33.27	
JMX-FXL2	15,071,992	2,260,798,800	0.55	94.77	88.73	32.2	
JMX-ZMK1	11,275,042	1,691,256,300	0.61	94.06	87.64	31.98	
JMX-ZS-1	13,468,744	2,020,311,600	0.55	94.8	88.83	31.98	
JXND-1	11,672,376	1,750,856,400	0.35	96.91	94	33.51	
JY-YCZ-1ys	9,748,242	1,462,236,300	0.5	95.76	91.47	33.1	
KC-DT-1	18,208,542	2,731,281,300	0.62	94.06	87.47	32.97	
KC-DT-4	13,493,348	2,024,002,200	0.58	94.47	88.22	33.08	
KC-MCH-1	17,596,514	2,639,477,100	0.61	94.12	87.53	32.9	
KC-MCH-2	12,397,002	1,859,550,300	0.46	95.72	89.86	33.22	
KW-DZ	13,738,748	2,060,812,200	0.47	95.65	89.73	32.95	
LD-LPSF-1y2	9,357,522	1,403,628,300	0.51	95.68	91.41	32.98	
LT-1-1	11,230,260	1,684,539,000	0.56	95.22	90.58	33.47	
M-10	11,973,734	1,796,060,100	0.28	97.7	95.42	33.03	
MEK-1	15,438,922	2,315,838,300	0.28	97.66	96.4	33.53	
MJ-1-1	9,933,684	1,490,052,600	0.54	95.39	90.89	33.98	
MJQ-DC2-1	9,825,574	1,473,836,100	0.49	95.83	91.61	33.49	
MJQ-DL-1	10,750,166	1,612,524,900	0.28	97.78	95.7	33.26	
MJQ-TGZ2	13,221,244	1,983,186,600	0.45	95.73	92.8	33.52	
ML-1-1	10,616,342	1,592,451,300	0.53	95.54	91.11	33.29	
MXB-1-1Y	35,459,252	5,354,347,052	0.42	96.14	90.73	33.56	
MXNC-1-Y	12,931,490	1,939,723,500	0.28	97.8	95.73	33.74	
MXNC-3-1Y	35,156,270	5,308,596,770	0.44	95.94	90.32	34.71	
MY-SLT-2	17,273,266	2,590,989,900	0.59	94.35	88.12	33.51	
MZ-1-1	15,426,650	2,313,997,500	0.28	97.8	95.73	33.99	
NN-1-1	9,575,538	1,436,330,700	0.57	95.08	90.38	33.26	
NQ-1-1	9,678,602	1,451,790,300	0.52	95.56	91.17	33.03	
NY-HZB-1	11,875,984	1,781,397,600	0.55	94.83	88.9	32.94	
PN-SG-1b	14,231,636	2,134,745,400	0.45	95.74	90.25	33.2	
PN-SG-1y	12,941,374	1,941,206,100	0.43	95.92	90.38	32.73	
PN-SG-2b	16,710,442	2,506,566,300	0.44	95.81	90.33	33.06	
PN-SG-3b	14,101,240	2,115,186,000	0.57	94.48	87.64	32.66	
PN-SG-3y	16,800,722	2,520,108,300	0.42	96.03	90.78	32.78	
SB-QQ-1	14,874,932	2,231,239,800	0.56	94.71	88.84	32.69	
SQ-1-3	38,560,676	5,822,662,076	0.42	96.08	90.62	33.87	
SR-FJA-1	11,094,454	1,664,168,100	0.36	96.81	93.81	33.72	
SR-FJA-2	11,201,130	1,680,169,500	0.35	96.92	94.02	33.22	
SR-HX-1	12,289,686	1,843,452,900	0.36	96.75	93.69	33.33	
ST-KYZ-1	12,656,350	1,898,452,500	0.34	97.03	94.22	33.27	
ST-SC-1	11,495,070	1,724,260,500	0.35	96.85	93.89	33.05	
TC-AD-1	13,093,764	1,964,064,600	0.44	95.88	90.3	32.63	
WN-LJ-2	14,137,924	2,120,688,600	0.45	95.81	90.14	33.03	
WYS-DWA-1	16,573,792	2,486,068,800	0.29	97.64	96.37	34.33	
WYS-LHF-1	12,060,454	1,809,068,100	0.35	96.89	93.97	33.34	
WYS-TZZ-1	10,992,050	1,648,807,500	0.41	96.14	92.29	32.44	
WYS-XFJL-1	10,774,304	1,616,145,600	0.34	96.97	94.11	32.88	
XH-DN-1	13,856,322	2,078,448,300	0.57	94.58	88.56	33.34	
XHS-1-1	11,247,846	1,687,176,900	0.52	95.56	91.17	34.02	
XS-LHK-1	12,733,488	1,910,023,200	0.57	94.56	88.55	32.05	
YL-TYD-1	9,574,510	1,436,176,500	0.34	96.96	94.1	33.51	
YL-TYD-2	12,756,758	1,913,513,700	0.33	97.12	94.39	33.85	
YX-PK-1	10,807,254	1,621,088,100	0.35	96.91	94	33.29	
YX-YS-2	11,584,796	1,737,719,400	0.33	97.14	94.43	33.32	
YY-NEY-1	14,245,298	2,136,794,700	0.56	94.58	88.36	32.09	
YY-TG-1	17,801,422	2,670,213,300	0.56	94.65	88.78	32.13	
ZZ-1-1y	31,145,950	4,703,038,450	0.43	95.93	90.31	33.84	
ZZ-2-1y	14,068,752	2,110,312,800	0.42	96.61	92.48	33.53	
AC-1	24,977,390 	3,746,608,500 	0.46 	97.67 	93.50 	33.36 	
AC-10	23,828,954 	3,574,343,100 	0.43 	97.99 	94.23 	33.49 	
AC-11	23,652,572 	3,547,885,800 	0.44 	97.83 	93.86 	33.13 	
AC-12	24,473,524 	3,671,028,600 	0.44 	97.94 	94.11 	33.08 	
AC-13	23,020,406 	3,453,060,900 	0.64 	95.84 	89.66 	32.41 	
AC-14	23,800,842 	3,570,126,300 	0.43 	97.96 	94.16 	33.00 	
AC-15	21,911,944 	3,286,791,600 	0.42 	98.06 	94.41 	33.26 	
AC-16	26,149,972 	3,922,495,800 	0.43 	97.99 	94.30 	34.99 	
AC-17	26,059,200 	3,908,880,000 	0.45 	97.72 	93.57 	33.13 	
AC-18	26,055,872 	3,908,380,800 	0.43 	97.96 	94.14 	33.03 	
AC-19	22,919,300 	3,437,895,000 	0.46 	97.70 	93.53 	33.55 	
AC-2	20,925,686 	3,138,852,900 	0.42 	98.08 	94.49 	33.57 	
AC-20	26,312,964 	3,946,944,600 	0.44 	97.83 	93.84 	32.79 	
AC-3	26,364,550 	3,954,682,500 	0.42 	98.06 	94.41 	33.24 	
AC-4	26,903,182 	4,035,477,300 	0.45 	97.75 	93.71 	33.61 	
AC-5	22,998,918 	3,449,837,700 	0.43 	98.02 	94.30 	33.91 	
AC-6	20,771,536 	3,115,730,400 	0.44 	97.82 	93.88 	33.38 	
AC-7	22,910,598 	3,436,589,700 	0.43 	98.00 	94.30 	33.59 	
AC-8	23,921,356 	3,588,203,400 	0.43 	98.03 	94.37 	33.43 	
AC-9	28,087,142 	4,213,071,300 	0.44 	97.82 	93.86 	33.14 	
