# Supplementary material for: Genomic analysis of Asian honeybee populations in China reveals evolutionary relationships and adaptation to abiotic stress
Source: Ecol Evol. 2020 Nov 2;10(23):13427–38. doi: 10.1002/ece3.6946 (PMC7713975; doi:10.1002/ece3.6946)
Supplement: Supplementary file 5 — Table S4 [file ECE3-10-13427-s005.docx]

­

Table S4. The information of effective reads matching the reference genome as well as geographic location for all the samples

| **Samples** | **Mapping rate(%)** | **Coverage(%)** | **Effective depth** |
| --- | --- | --- | --- |
| AB_mek1 | 80.39 | 98.73 | 10.01 |
| AB-1 | 87.21 | 98.91 | 7.71 |
| AB-10 | 87.57 | 99.11 | 8.96 |
| AB-11 | 88.71 | 99.39 | 11.89 |
| AB-12 | 87.76 | 99.12 | 8.39 |
| AB-13 | 86.62 | 98.89 | 7.92 |
| AB-14 | 88.31 | 99.19 | 11.04 |
| AB-15 | 87.01 | 98.81 | 7.86 |
| AB-16 | 86.53 | 99.34 | 9.52 |
| AB-2 | 88.39 | 99.04 | 8.27 |
| AB-3 | 89.01 | 99.1 | 8.65 |
| AB-4 | 87.61 | 99.29 | 9.26 |
| AB-5 | 87.64 | 99.37 | 9.75 |
| AB-6 | 87.98 | 99.09 | 10.07 |
| AB-7 | 87.59 | 99.37 | 10.44 |
| AB-8 | 86.81 | 99.48 | 11.7 |
| AB-9 | 87.54 | 99.23 | 9.52 |
| MEK-1 | 88.86 | 99.01 | 9.68 |
| AT-CBQ-1 | 94.18 | 99.73 | 13.04 |
| AT-SJZ-1 | 93.75 | 99.71 | 12.12 |
| AT-SJZ-2 | 94.92 | 99.58 | 10.84 |
| AT-SJZ-3 | 94.48 | 98.27 | 7.27 |
| AC-19 | 94.28 | 99.93 | 14.77 |
| AC-20 | 94.42 | 99.95 | 16.98 |
| B-2 | 87.55 | 98.12 | 6.61 |
| B-3 | 86.66 | 97.45 | 5.95 |
| B-3-1 | 86.97 | 99.11 | 8.33 |
| B-3-2 | 87.24 | 99.27 | 9.1 |
| B-4 | 87.3 | 97.73 | 6.27 |
| B-4-1 | 87.1 | 99.04 | 8.41 |
| B-4-2 | 87.93 | 98.91 | 7.96 |
| B-5 | 89.24 | 98.57 | 7.24 |
| B-5-1 | 87.54 | 99.09 | 8.5 |
| B-5-2 | 87.52 | 98.93 | 8.02 |
| M-10 | 86.77 | 98.57 | 7.31 |
| BS-LL-1 | 87.66 | 99.19 | 8.61 |
| BS-LZ-1 | 89.02 | 99.14 | 8.89 |
| BS-LZ-2 | 87.15 | 99.19 | 8.3 |
| BS-LZ-4 | 87.4 | 98.98 | 7.83 |
| BS-LZ-6 | 87.7 | 99.26 | 8.37 |
| TC-AD-1 | 87.41 | 99.17 | 8.05 |
| WN-LJ-2 | 87.42 | 99.25 | 8.69 |
| DA-LK-1 | 86.74 | 98.82 | 7.49 |
| ML-1-1 | 86.79 | 98.45 | 6.47 |
| MXB-1-1Y | 87.99 | 99.66 | 22.12 |
| MXNC-1-Y | 86.23 | 99.13 | 7.85 |
| MXNC-3-1Y | 88.17 | 99.59 | 21.96 |
| MZ-1-1 | 86.64 | 99.33 | 9.39 |
| NN-1-1 | 87.05 | 97.32 | 5.86 |
| NQ-1-1 | 86.5 | 97.8 | 5.88 |
| XHS-1-1 | 88.37 | 97.54 | 7.01 |
| DDG-1-2Y | 88.85 | 96.26 | 6.47 |
| DY-1-1 | 87.57 | 98.38 | 6.57 |
| LT-1-1 | 86.91 | 98.03 | 6.86 |
| MJ-1-1 | 87.74 | 97.8 | 6.12 |
| WYS-DWA-1 | 89.1 | 99.12 | 10.43 |
| WYS-LHF-1 | 86.85 | 98.82 | 7.39 |
| WYS-TZZ-1 | 87.89 | 98.64 | 6.8 |
| WYS-XFJL-1 | 86.97 | 98.53 | 6.6 |
| YX-PK-1 | 87.98 | 98.45 | 6.69 |
| YX-YS-2 | 88.46 | 98.46 | 7.22 |
| ST-KYZ-1 | 87.12 | 98.91 | 7.76 |
| ST-SC-1 | 88.36 | 98.72 | 7.16 |
| SR-FJA-1 | 85.59 | 98.34 | 6.69 |
| SR-FJA-2 | 86.37 | 98.59 | 6.81 |
| GNJ-XD-1 | 86.31 | 97.84 | 5.86 |
| YL-TYD-1 | 88.04 | 97.35 | 5.94 |
| YL-TYD-2 | 88.79 | 98.54 | 7.99 |
| JGS-LJP-1 | 87.39 | 98.62 | 7.5 |
| JGS-LZ-1 | 87.63 | 98.31 | 6.78 |
| JXND-1 | 87.37 | 98.69 | 7.18 |
| FJS-LJB | 87.19 | 99.08 | 7.6 |
| KC-MCH-1 | 86.26 | 99.42 | 8.94 |
| KC-MCH-2 | 88.08 | 99.14 | 7.69 |
| KW-DZ | 86.35 | 99.26 | 8.34 |
| YY-NEY-1 | 86.64 | 99.07 | 7.27 |
| YY-TG-1 | 86.16 | 99.44 | 9.04 |
| AC-5 | 94.45 | 99.86 | 14.89 |
| AC-6 | 93.48 | 99.94 | 13.25 |
| AC-13 | 93.12 | 99.95 | 14.68 |
| AC-14 | 93.32 | 99.95 | 15.19 |
| AC-15 | 94 | 99.94 | 14.11 |
| AC-16 | 93.75 | 99.26 | 16.87 |
| AC-17 | 93.62 | 99.96 | 16.65 |
| AC-18 | 93.63 | 99.97 | 16.67 |
| FS-TH-1 | 87.74 | 99.17 | 8.55 |
| SB-QQ-1 | 87.17 | 99.13 | 7.65 |
| XH-DN-1 | 87.22 | 98.87 | 7.13 |
| XS-LHK-1 | 86.35 | 98.72 | 6.48 |
| NY-HZB-1 | 86.49 | 98.27 | 6.06 |
| MY-SLT-2 | 86.88 | 99.21 | 8.84 |
| HP-HH-1 | 86.63 | 99.25 | 8.34 |
| HB_jm1 | 87.96 | 98.32 | 9.65 |
| HB_jm2 | 90.08 | 97.56 | 7.95 |
| AC-1 | 93.11 | 99.95 | 15.87 |
| CK_bslm1 | 87.59 | 97.76 | 8.25 |
| CK-bs | 87.23 | 99.05 | 7.69 |
| CK-bslm2 | 81.34 | 98.82 | 6.95 |
| CK-daxl | 84.63 | 99.11 | 8.56 |
| CK-hab | 77.66 | 98.93 | 8.65 |
| CK-qq | 87.93 | 99.66 | 21.7 |
| SQ-1-3 | 88.06 | 99.68 | 24.03 |
| ZZ-1-1y | 88.89 | 99.63 | 19.63 |
| PN-SG-1b | 87.66 | 99.24 | 8.77 |
| PN-SG-1y | 87.52 | 99.06 | 7.97 |
| PN-SG-2b | 86.52 | 99.46 | 10.15 |
| PN-SG-3b | 87.18 | 99.22 | 8.64 |
| PN-SG-3y | 87.18 | 99.43 | 10.3 |
| ZZ-2-1y | 88.14 | 98.42 | 8.73 |
| FP-XMG-2 | 88.17 | 99.3 | 8.71 |
| MJQ-DC2-1 | 88.04 | 97.88 | 6.1 |
| MJQ-DL-1 | 87.95 | 98.68 | 6.66 |
| MJQ-TGZ2 | 86.69 | 99.16 | 8.05 |
| LD-LPSF-1y2 | 86.08 | 97.83 | 5.63 |
| SR-HX-1 | 86.98 | 98.89 | 7.52 |
| CS-JS-2 | 87 | 99.23 | 9.58 |
| CS-JSTZY | 87.11 | 99.32 | 8.34 |
| HB-LC-1 | 84.9 | 99.18 | 8.06 |
| HC-1 | 91.54 | 99.32 | 9.28 |
| JMX-FXL2 | 86.77 | 99.2 | 7.71 |
| JMX-ZMK1 | 85.91 | 98.18 | 5.7 |
| JMX-ZS-1 | 86.43 | 98.83 | 6.86 |
| JY-YCZ-1ys | 86.9 | 97.86 | 5.96 |
| KC-DT-1 | 86.23 | 99.44 | 9.26 |
| KC-DT-4 | 85.01 | 98.73 | 6.75 |
| AC-10 | 93.37 | 99.92 | 15.2 |
| AC-11 | 93.5 | 99.95 | 15.12 |
| AC-12 | 94.14 | 99.96 | 15.73 |
| AC-2 | 92.36 | 99.93 | 13.19 |
| AC-3 | 93.97 | 99.95 | 16.93 |
| AC-4 | 92.47 | 99.96 | 16.94 |
| AC-7 | 93.82 | 99.94 | 14.67 |
| AC-8 | 93.74 | 99.96 | 15.29 |
| AC-9 | 93.94 | 99.97 | 17.99 |
| HLOLZ-1-2 | 88.52 | 97.63 | 7.48 |
| HLO-YFJ1 | 86.71 | 99.19 | 8.07 |
| HLQC-1-1 | 87.06 | 98.46 | 6.53 |
| JC-1-1 | 87.22 | 98.6 | 7.34 |

*Mapping rates mean the proportion of the numbers of reads mapping the genome occupied the total numbers of reads; Coverage means the proportion of the genomic region covered by reads occupied the whole genomic region; depth means that, on average, each base of genome has been sequenced a certain number of time.
