# Supplementary material for: Genomic analysis of Asian honeybee populations in China reveals evolutionary relationships and adaptation to abiotic stress
Source: Ecol Evol. 2020 Nov 2;10(23):13427–38. doi: 10.1002/ece3.6946 (PMC7713975; doi:10.1002/ece3.6946)
Supplement: Supplementary file 6 — Table S5 [file ECE3-10-13427-s006.docx]

Table S5 PCR primers used for generation of CNAG_01904Δ and complementation in this study.

| Primers | Targeted genes | Sequence(5-3) |
| --- | --- | --- |
| TL17 | M13 F | GGGCGCCCGGTTCTTTTTGTCA |
| TL18 | M13 R | GGGCGCCCGGTTCTTTTTGTCA |
| TL19 | NEO split primers F | GGGCGCCCGGTTCTTTTTGTCA |
| TL20 | NEO split primers R | TTGGTGGTCGAATGGGCAGGTAGC |
| TL59 | NEO reverse primer | TGTGGATGCTGGCGGAGGATA |
| TL1043 | CNAG_01094 KO F1 | TGCCCTTTTCCTTATGGTGTTTGGTA |
| TL1044 | CNAG_01094 KO R1 | CTGGCCGTCGTTTTACGTGGCTGATGTATGGGGAGGGTAGAG |
| TL1045 | CNAG_01094 KO F2 | GTCATAGCTGTTTCCTGATTACCCGCTTTTTGGACCGAGTG |
| TL1046 | CNAG_01094 KO R2 | GGCCAGATATCTAGTGCGACAAGT |
| TL1047 | CNAG_01094 KO F3 | TGCGCATTTTCAGGGGTAACG |
| TL1048 | CNAG_01094 KO R3 | CACGGGTAAAGGCAGGTATGAAGAAC |
| TL1049 | CNAG_01094 KO F4 | AGGGCTTGATGGTGGTGGAGTAGATG |
| TL1075 | APICC_05210 OE F | CGCCCAACATGTCTGGATCCATGACTCATTTTCGCGGATTCTAT |
| TL1076 | APICC_05210 OE R | ACGTCGTATGGGTAGGATCCAAACGGTAAACCTATTTCAAAGGC |
