# Supplementary material for: Genomic analysis of Asian honeybee populations in China reveals evolutionary relationships and adaptation to abiotic stress
Source: Ecol Evol. 2020 Nov 2;10(23):13427–38. doi: 10.1002/ece3.6946 (PMC7713975; doi:10.1002/ece3.6946)
Supplement: Supplementary file 7 — Table S6 [file ECE3-10-13427-s007.docx]

| **Sample** | **Variable SNPs** | **Pi** | **θw** | **Numbers of samples** |
| --- | --- | --- | --- | --- |
| Diannan | 3052652 | 0.002513 | 0.003809 | 12 |
| WSichPl | 2579347 | 0.002225 | 0.002897 | 18 |
| ChangMt | 1289366 | 0.001871 | 0.001889 | 6 |
| TibetPl | 1701602 | 0.002105 | 0.002175 | 14 |
| HainanId | 2263961 | 0.002431 | 0.003067 | 5 |
| TaiLvMt | 2635272 | 0.002853 | 0.00357 | 13 |
| **CentCh** |  |  |  |  |
| ZheFuH | 2742459 | 0.002422 | 0.003506 | 11 |
| WuMt | 2072721 | 0.002575 | 0.003198 | 6 |
| YiMt | 2812227 | 0.002929 | 0.003949 | 8 |
| ShenFr | 2566439 | 0.002519 | 0.003477 | 9 |
| DaMt | 2825652 | 0.002755 | 0.003828 | 9 |
| Others | 5558562 | 0.009962 | 0.013147 | 27 |
| Totals | 9166395 | 0.002681 | 0.006778 | 138 |

**Table S6 Genetic variation and population diversity**
