# Supplementary material for: Genomic analysis of Asian honeybee populations in China reveals evolutionary relationships and adaptation to abiotic stress
Source: Ecol Evol. 2020 Nov 2;10(23):13427–38. doi: 10.1002/ece3.6946 (PMC7713975; doi:10.1002/ece3.6946)
Supplement: Supplementary file 9 — Table S8 [file ECE3-10-13427-s009.docx]

| Term | ID | Input number | Background number | P-Value | Corrected  P-Value | Input |
| --- | --- | --- | --- | --- | --- | --- |
| Hippo signaling pathway - fly | dme04391 | 4 | 61 | 0.001529142 | 0.044244113 | APICC_02410\|APICC_09504\|APICC_07309\|APICC_04160 |
| Biosynthesis of amino acids | dme01230 | 4 | 66 | 0.002011096 | 0.044244113 | APICC_02894\|APICC_07023\|APICC_09661\|APICC_02409 |
| Citrate cycle (TCA cycle) | dme00020 | 3 | 43 | 0.005186228 | 0.07606467 | APICC_02894\|APICC_07624\|APICC_09661 |
| Glycolysis / Gluconeogenesis | dme00010 | 3 | 55 | 0.009866807 | 0.080905457 | APICC_07023\|APICC_07624\|APICC_02409 |
| Neuroactive ligand-receptor interaction | dme04080 | 3 | 56 | 0.010338346 | 0.080905457 | APICC_06681\|APICC_06768\|APICC_08806 |
| 2-Oxocarboxylic acid metabolism | dme01210 | 2 | 19 | 0.011032562 | 0.080905457 | APICC_02894\|APICC_09661 |
| Apoptosis - multiple species | dme04215 | 2 | 23 | 0.01545113 | 0.089499388 | APICC_07309\|APICC_00924 |
| Carbon metabolism | dme01200 | 4 | 123 | 0.016272616 | 0.089499388 | APICC_02894\|APICC_07023\|APICC_09661\|APICC_02409 |
| Glyoxylate and dicarboxylate metabolism | dme00630 | 2 | 33 | 0.029167766 | 0.142597969 | APICC_02894\|APICC_09661 |
| MAPK signaling pathway - fly | dme04013 | 3 | 93 | 0.037122162 | 0.160376438 | APICC_02410\|APICC_07309\|APICC_08320 |
| mTOR signaling pathway | dme04150 | 3 | 96 | 0.040094109 | 0.160376438 | APICC_05658\|APICC_04380\|APICC_04160 |
| Glycerolipid metabolism | dme00561 | 2 | 42 | 0.044369422 | 0.162687881 | APICC_04451\|APICC_00436 |

Table S8. The enrichment analysis of adaptive-selected genes in KEGG pathway
